# Sesquiterpene lactones from *Artemisia vulgaris* L. as potential NO inhibitors in LPS-induced RAW264.7 macrophage cells

**DOI:** 10.3389/fchem.2022.948714

**Published:** 2022-09-01

**Authors:** Xiang-Yu Chen, Tao Liu, Yu-Ze Hu, Tian-Tian Qiao, Xiu-Juan Wu, Ping-Hua Sun, Chui-Wen Qian, Zhe Ren, Jun-Xia Zheng, Yi-Fei Wang

**Affiliations:** ^1^ Department of Cell Biology, College of Life Science and Technology, Jinan University, Guangzhou, China; ^2^ Guangdong Province Key Laboratory of Bioengineering Medicine, Guangzhou, China; ^3^ Guangdong Provincial Biotechnology Drug & Engineering Technology Research Center, Guangzhou, China; ^4^ National Engineering Research Center of Genetic Medicine, Guangzhou, China; ^5^ College of Pharmacy, Jinan University, Guangzhou, China; ^6^ GuangZhou (Jinan) Biomedical Research and Development Center Co., Ltd., Guangzhou, China; ^7^ School of Biomedical and Pharmaceutical Sciences, Guangdong University of Technology, Guangzhou, China

**Keywords:** *Artemisia vulgaris* L., new structures, guaianolide sesquiterpene lactones, anti-inflammatory, *in silico*

## Abstract

Twelve new guaianolide sesquiterpene lactones (**1**–**12**), along with ten known analogs (**13**–**22**) were isolated from an EtOH extract of the dried aerial parts of *Artemisia vulgaris* L. The new structures were elucidated via abundant spectroscopic data analyses (HRESIMS, IR, 1D, and 2D NMR), and the absolute configurations of these compounds were determined by X-ray crystallography and ECD calculations. The compounds (**1**−**22**) were identified as guaiane-type sesquiterpenes with characteristic α-methylene-γ-lactone and α,β-unsaturated carbonyl moieties. All compounds were tested for their inhibitory activity against NO production in lipopolysaccharide-stimulated RAW264.7 macrophages. The isolated sesquiterpenoids dose-dependently exhibited an NO production inhibitory activity by inhibiting the expression of inducible NO oxidase (iNOS) and cyclooxygenase-2 (COX-2) with IC_50_ values ranging from 1.0 to 3.6 μM. The inhibitory effect on the NO production of the compounds (**1**–**4** and **6**–**22**) is better than that of the positive control (dexamethasone). The different substitutions of compounds on C-8 influence anti-inflammatory effects, as evidenced by the *in silico* analysis of related binding interactions of new compounds (**1**–**12**) with iNOS.

## 1 Introduction

*Artemisia vulgaris* L. (mugwort), belonging to the family of Asteraceae, is widespread throughout Asia, North America, and Europe (Weston et al., 2005). *A. vulgaris*, as a kind of traditional medicinal plant, has been extensively used for relieving pain and treating gynecological symptoms in folks (Hershoff, 2001; Pires et al., 2009). The chemical constituents of *A. vulgaris* contain mainly polysaccharides, flavonoids, terpenoids, and sterols, showing anti-tumor, anti-inflammatory, hepatoprotective, anti-oxidant, immunomodulatory, anti-allergic, and anti-bacterial activities (Schmid-Grendelmeier et al., 2003; Blagojević et al., 2006; Saleh et al., 2014; Abiri et al., 2018; Soon et al., 2019; Ekiert et al., 2020). Previous phytochemical investigations reported that sesquiterpenoids are among the most critical ingredients of secondary metabolites in the genus *Artemisia* and have strong application values in contemporary medicine, food, and the perfume industry (Duke and Bogenschutz, 1994; Bora and Sharma, 2011; Rasheed et al., 2017; Sundararajan and Kumari, 2017). It is widely known that artemisinin and arglabin are promising potent remedies in the treatment of malaria and cancer (Lone et al., 2015; Kumari et al., 2019). *A. vulgaris* and *Artemisia annua* L*.* are very similar in many aspects (Funk, 2009). Therefore, *A. vulgaris* is regarded as an abundant producer of biologically effective sesquiterpene lactones, prompting us to select it for a detailed study.

Inflammation is an essential immune response to pathogens, toxins, and local injuries. The beneficial effects in the physiological or acute inflammation might turn deleterious in a persistent or over-inflammatory response. Macrophage is a primary cell type in directing the host’s inflammatory and immune processes, and the excessive release of nitric oxide is an essential sign of an inflammatory response. Testing the inhibitory effect of compounds on NO’s release in macrophages is an integral approach for revealing novel small molecules with anti-inflammatory activities. Our investigation for architecturally unique and effective chemical constituents from *A. vulgaris* led to the isolation and structural elucidation of 22 compounds, including 12 new sesquiterpenoids. And, all the compounds were assessed for anti-inflammatory effects by LPS-induced RAW264.7 macrophage cells.

## 2 Materials and methods

### 2.1 General experimental procedures

UV spectra were attained on a JASCO V-730 spectrophotometer. IR data were obtained on a Nicolet iS50 spectrometer (Thermo Fisher Scientific, United States) using KBr pellets. Optical rotations were measured using a JASCO P-2000 polarimeter at room temperature. HRESIMS spectra were recorded with an AB Sciex Triple-TOF 5600+ apparatus. ECD measurements were conducted on a Chirascan Plus spectrometer (Applied Photophysics, United Kingdom). 1D and 2D NMR spectra were obtained on a Bruker AVANCE III 500 or 600 MHz instruments. Chemical shifts were reported in ppm (δ) with coupling constants (J) in hertz. The residual signals of CDCl_3_ were used as references. Single crystal X-ray diffraction (Rigaku Oxford Diffraction Supernova Dual Source) was used to measure the crystal structures. Silica gel (100–200 and 200–300 meshes, Qingdao Marine Chemical Co., Ltd., China), Sephadex LH-20 gel (GE Healthcare, Sweden), YMC ODS-A-HG gel (50 μm, YMC, Japan), and MCI gel (SaiPuRuiSi. Beijing, China) were used to perform column chromatography (CC). Semi-Preparative HPLC was performed on a Wufeng LC-100 apparatus (Shanghai Wufeng Co., Ltd., China) using photodiode array (PDA) UV analysis at 210 nm with a YMC-Pack ODS-A column (250 × 10 mm, 5 μm, 3 ml/min).

### 2.2 Plant material

The whole plant of *A. vulgaris* was collected from Tangyin city, Henan Province, China, in May 2018 and authenticated by Yifei Wang (College of Life Science and Technology, Jinan University). A specimen (No. 201805) was deposited in Guangzhou Jinan Biomedicine Research and Development Center, Jinan University.

### 2.3 Extraction and isolation

After drying and grinding, the plant sample (40.0 kg) of *A. vulgaris* was extracted with 95% EtOH at room temperature (360 L, 3 times) in order to afford a residue (3.6 kg), which was suspended in H_2_O and extracted with EtOAc and PE (8 L, 3 times) to get an EtOAc partition and a PE partition, successively. The EtOAc partition (1.1 kg) was applied to the MCI gel column using gradient mixtures (10 × 150 cm, H_2_O-MeOH, 60:40 to 0:100), yielding 9 fractions (Fr. 1-9). Fr. 7 (120.0 g) was subsequently chromatographed on a silica gel column (100–200 mesh, 10 × 47 cm, EtOAc-PE, from 12:88 to 100:0) to afford five subfractions (Fr. 7.1–7.5). Fr. 7-2 was separated by Sephadex LH-20 column (2 × 110 cm, MeOH-CH_2_Cl_2_, 50:50) to remove pigments and obtain Fr. 7.2.1 and Fr. 7.2.2. Fr. 7.2.2 (5.7 g) was passed through an opening ODS CC (5.0 × 17.5 cm, H_2_O-MeOH, 50:50–100:0), and the afforded fractions were divided into Fr. 7.2.2.1–7.2.2.14, as instructed by the TLC analysis. The 14 fractions were analyzed on the HPLC-MS, and all were identified to be separated on a silica gel column (200–300 mesh, 2.5 × 5.5 cm) eluted with a stepwise gradient of EtOAc-PE (5:95 to 100:0) in the next step. Subsequently, further isolation of Fr. 7.2.2.10 (503.0 mg) on Si CC provided four subfractions (Fr. 7.2.2.10a–7.2.2.10d). The obtained Fr. 7.2.2.10b (377.0 mg) was purified by semi-preparative HPLC (H_2_O-MeCN, 60:40, flow rate: 3.0 ml min^−1^) to yield compounds **1** (36.5 mg, t_R_ = 69.0 min), **2** (22.6 mg, t_R_ = 74.9 min), and **3** (28.5 mg, t_R_ = 80.4 min). Fr. 7.2.2.9 (433.0 mg) was subjected to Si CC to obtain four subfractions (Fr. 7.2.2.9a-7.2.2.9d). The obtained Fr. 7.2.2.9b (237.0 mg) was further purified by semi-preparative HPLC (H_2_O-MeOH, 40:60, 3.0 ml min^−1^) to give compounds **7** (12.7 mg, t_R_ = 42.9 min), **13** (36.9 mg, t_R_ = 46.8 min), and **4** (27.0 mg, t_R_ = 49.5 min). Fr. 7.2.2.6 (483.0 mg) was subjected to Si CC to obtain six subfractions (Fr. 7.2.2.6a–7.2.2.6f). The obtained Fr. 7.2.2.6d (55.0 mg) was further purified by semi-preparative HPLC (H_2_O-MeCN, 59:41, 3.0 ml min^−1^) to give compound **18** (13.2 mg, t_R_ = 50.0 min). Using similar separation procedures, **17** (4.1 mg, t_R_ = 37.8 min), **6** (26.4 mg, t_R_ = 39.2 min), and **16** (9.2 mg, t_R_ = 57.2 min) were obtained from Fr. 7.2.2.6e (149.0 mg). Fr. 7.2.2.7 (452.3 mg) was subjected to Si CC eluted by EtOAc-PE to yield Fr. 7.2.2.7a–7.2.2.7d. Fr. 7.2.2.7b (138.8 mg), which was purified by semi-preparative HPLC (H_2_O-MeCN, 55:45, 3.0 ml min^−1^) to afford compound **15** (10.4 mg, t_R_ = 53.0 min) and Fr. 7.2.2.7b.1 (19.8 mg). Compound **5** (2.4 mg, t_R_ = 38.9 min) was further obtained from Fr. 7.2.2.7b.1 by semi-preparative HPLC (H_2_O-MeOH, 40:60, 3.0 ml min^−1^). In a similar way, Fr. 7.2.2.8 (542.1 mg) was treated with an Si CC yielding six sections (Fr. 7.2.2.8a–7.2.2.8f), and finally semi-preparative HPLC (H_2_O-MeCN, 59:41, 3.0 ml min^−1^) to generate compound **14** (18.4 mg, t_R_ = 51.9 min) from Fr. 7.2.2.8d (100.2 mg). Compound **19** (16.3 mg) was precipitated from Fr. 7.2.2.8e (140.7 mg) in the form of crystals. Fr. 7.2.2.2 (300.1 mg) was then separated on Si CC to give Fr. 7.2.2.2a–7.2.2.2c. Compound **20** (18.0 mg, t_R_ = 50.0 min) was obtained from Fr. 7.2.2.2b (181.0 mg) and **22** (12.1 mg, t_R_ = 57.3 min) was obtained from Fr. 7.2.2.2c (31.8 mg) via semi-preparative HPLC (H_2_O-MeCN, 71:29, 3.0 ml min^−1^). Fr. 7.2.2.3 (241.8 mg) was subjected to Si CC to obtain two subfractions (Fr. 7.2.2.3a–7.2.2.3b). Fr. 7.2.2.3b (154.4 mg) was further subjected to semi-preparative HPLC (H_2_O-MeCN, 67:33, 3.0 ml min^−1^) to afford **11** (4.3 mg, t_R_ = 46.7 min) and **21** (16.9 mg, t_R_ = 52.1 min). Fr. 7.2.2.4 (204.4 mg) was separated by Si CC to give five subfractions (Fr. 7.2.2.4a–7.2.2.4e), and Fr. 7.2.2.4c (57.3 mg) was purified by semi-preparative HPLC (H_2_O-MeCN, 62:38, 3.0 ml min^−1^), yielding **9** (5.1 mg, t_R_ = 39.0 min) and **10** (2.8 mg, t_R_ = 44.7 min). Compound **12** (12.0 mg, t_R_ = 35.2 min) was obtained from Fr. 7.2.2.4b (77 mg) by semi-preparative HPLC (H_2_O-MeCN, 62:38, 3.0 ml min^−1^). Fr. 7.2.2.5 (370.0 mg) was further subjected to CC over silica gel to yield four subfractions, followed by purification with semi-preparative HPLC (H_2_O-MeCN, 60:40, 3.0 ml min^−1^) to afford **8** (19.8 mg, t_R_ = 54.6 min).

#### 2.3.1 Artemvulactone H (**1**)

Colorless oil; [α]_D_^20^ + 142 (c 0.125, MeOH); UV (MeOH) *λ*_max_ (log *ε*) 208 (3.64) nm; IR (KBr) *ν*_max_/cm^−1^: 3,432, 2,925, 1,767, 1,711, 1,643, 1,380, 1,273, 1,229, 1,145, 1,004. ^1^H and ^13^C NMR data (Tables 1, 3); HRESIMS *m/z* 367.1504 [M + Na]^+^ (calcd for C_20_H_24_O_5_Na, 367.1516).

**TABLE 1:** ^1^H NMR spectroscopic data for compounds **1–7**.

No.	1^a^	2^b^	3^a^	4^a^	5^a^	6^b^	7^a^
	*δ*_H_ (J in Hz)	*δ*_H_ (J in Hz)	*δ*_H_ (J in Hz)	*δ*_H_ (J in Hz)	*δ*_H_ (J in Hz)	*δ*_H_ (J in Hz)	*δ*_H_ (J in Hz)
2a	2.65, m	2.63, m	2.62, m	2.31, m	2.46, m	1.93, d (15.3)	2.30, m
2b	–	–	–	2.89, d (17.0)	2.93, d (16.8)	2.24, dd (20.6, 9.4)	–
3	5.50, m	5.49, s	5.49, s	5.55, d (1.3)	5.54, s	3.58, s	5.57, s
5	2.85, d (10.7)	2.84, d (10.7)	2.82, d (10.7)	2.67, m	2.71, m	2.37, d (11.2)	2.71, dd (14.4, 5.6)
6	3.98, dd (10.7,9.2)	3.95, dd (10.7,9.2)	3.94, dd (10.6, 9.2)	3.88, m	3.87, t (9.7)	3.94, dd (11.0, 9.2)	3.92, dd (10.4, 9.4)
7	3.58, tt (10.2, 3.2)	3.54, tt (9.8, 3.2)	3.51, m	3.28, tt (9.7, 3.2)	2.46, m	3.27, m	3.33, tt (9.7, 3.2)
8	5.42, ddd (10.3, 3.6, 1.6)	5.34, ddd (10.3, 3.8, 1.6)	5.33, ddd (10.3, 3.6, 1.6)	4.94, ddd (10.3, 6.3, 4.9)	4.97, td (9.1, 4.6)	4.96, td (9.9, 2.1)	5.05, ddd (10.5, 6.0, 4.8)
9a	5.50, m	5.42, dd (3.8, 1.2)	5.45, dd (3.6, 1.2)	2.56, dd (13.9, 4.8)	2.57, dd (13.0, 4.5)	2.24, dd (20.6, 9.4)	2.59, dd (14.0, 4.7)
9b	–	–	–	2.67, m	2.71, m	2.58, dd (15.3, 2.6)	2.71, dd (14.4, 5.6)
11	–	–	–	–	2.46, m	–	–
13a	5.68, d (3.0)	5.71, d (3.0)	5.69, d (3.0)	5.63, d (3.0)	1.27, d (6.4)	5.62, d (3.0)	5.63, d (3.0)
13b	6.26, d (3.0)	6.27, d (3.0)	6.27, d (3.0)	6.22, d (3.0)	–	6.23, d (3.0)	6.21, m
14a	1.94, m	1.93, m	1.92, m	5.14, s	5.21, s	5.00, s	5.16, s
14b	–	–	–	5.40, s	5.31, s	5.55, s	5.44, s
15	1.94, m	1.93, m	1.92, m	1.91, m	1.90, d (11.4)	1.66, s	1.94, m
2′	–	2.43, m	2.27, m	2.31, m	–	2.63, m	–
3′a	–	1.50, m	2.15, m	2.15, m	6.17, q (7.3)	1.23, dd (8.7, 7.0)	6.21, m
3′b	6.21, qd (7.3, 1.4)	1.75, dt (13.7, 7.4)	–	–	–	–	–
4′	2.03, m	0.94, t (7.4)	0.99, d (6.6)	1.00, d (7.1)	2.03, d (7.2)	1.23, dd (8.7, 7.0)	2.04, dd (7.3, 1.5)
5′	1.94, m	1.21, d (7.0)	0.99, d (6.6)	1.00, d (7.1)	1.90, d (11.4)	–	1.94, m

a 500 MHz in CDCl_3_.

b 600 MHz in CDCl_3_.

#### 2.3.2 Artemvulactone I (**2**)

White powder; [α]_D_^20^ + 80 (c 0.251, MeOH); UV (MeOH) *λ*_max_ (log *ε*) 206 (3.53) nm; IR (KBr) *ν*_max_/cm^−1^: 3,465, 2,968, 1,769, 1,732, 1,660, 1,379, 1,273, 1,242, 1,144, 1,006. ^1^H and ^13^C NMR data (Tables 1, 3); HRESIMS *m/z* 369.1662 [M + Na]^+^ (calcd for C_20_H_26_O_5_Na, 369.1672).

#### 2.3.3 Artemvulactone J (**3**)

Colorless oil; [α]_D_^20^ + 75 (c 0.525, MeOH); UV (MeOH) *λ*_max_ (log *ε*) 207 (3.03) nm; IR (KBr) *ν*_max_/cm^−1^: 3,455, 2,959, 1,769, 1,735, 1,659, 1,370, 1,274, 1,245, 1,149, 1,005. ^1^H and ^13^C NMR data (Tables 1, 3); HRESIMS *m/z* 369.1664 [M + Na]^+^ (calcd for C_20_H_26_O_5_Na, 369.1672).

#### 2.3.4 Artemvulactone K (**4**)

Colorless oil; [α]_D_^20^ + 165 (c 0.387, MeOH); UV (MeOH) *λ*_max_ (log *ε*) 206 (3.82) nm; IR (KBr) *ν*_max_/cm^−1^: 3,460, 2,958, 1,769, 1,735, 1,655, 1,366, 1,293, 1,264, 1,147, 1,010. ^1^H and ^13^C NMR data (Tables 1, 3); HRESIMS *m/z* 347.1862 [M + H]^+^ (calcd for C_20_H_26_O_5_, 347.1853).

#### 2.3.5 Artemvulactone L (**5**)

Colorless oil; [α]_D_^20^ + 130 (c 0.132, MeOH); UV (MeOH) *λ*_max_ (log *ε*)216 (3.60) nm; IR (KBr) *ν*_max_/cm^−1^: 3,469, 2,940, 1,773, 1,712, 1,640, 1,379, 1,232, 1,157, 1,004. ^1^H and ^13^C NMR data (Tables 1, 3); HRESIMS *m/z* 369.1660 [M + Na]^+^ (calcd for C_20_H_26_O_5_Na, 369.1672).

#### 2.3.6 Artemvulactone M (**6**)

White powder; [α]_D_^20^ + 113 (c 0.18, MeOH); UV (MeOH) *λ*_max_ (log *ε*) 207 (3.65) nm; IR (KBr) *ν*_max_/cm^−1^: 3495,2975, 1772, 1731, 1387, 1268, 1144, 1075, 965. ^1^H and ^13^C NMR data (Tables 1, 3); HRESIMS *m/z* 371.1450 [M + Na]^+^ (calcd for C_19_H_24_O_6_Na, 371.1465).

#### 2.3.7 Artemvulactone N (**7**)

Colorless oil; [α]_D_^20^ + 158 (c 0.15, MeOH); UV (MeOH) *λ*_max_ (log *ε*) 208 (3.49) nm; IR (KBr) *ν*_max_/cm^−1^: 3,445, 2,925, 1,768, 1,713, 1,646, 1,454, 1,263, 1,235, 1,145, 1,008. ^1^H and ^13^C NMR data (Tables 1, 3); HRESIMS *m/z* 345.1703 [M + H]^+^ (calcd for C_20_H_24_O_5_, 345.1697).

#### 2.3.8 Artemvulactone O (**8**)

White powder; [α]_D_^20^ + 105 (c 0.215, MeOH); UV (MeOH) *λ*_max_ (log *ε*) 207 (3.50) nm; IR (KBr) *ν*_max_/cm^−1^: 3,476, 2,934, 1,769, 1,730, 1,658, 1,469, 1,275, 1,250, 1,146, 1,011. ^1^H and ^13^C NMR data (Tables 2, 3); HRESIMS *m/z* 371.1456 [M + Na]^+^ (calcd for C_19_H_24_O_6_Na, 371.1465).

**TABLE 2:** ^1^H NMR spectroscopic data for compounds **8–12**.

No.	8^a^	9^a^	10^a^	11^a^	12^b^
	*δ*_H_ (J in Hz)	*δ*_H_ (J in Hz)	*δ*_H_ (J in Hz)	*δ*_H_ (J in Hz)	*δ*_H_ (J in Hz)
2a	1.94, d (14.9)	—	—	1.94, d (16.0)	3.69, s
2b	2.47, d (15.0)	—	—	2.48, m	—
3	3.55, s	6.20, m	6.04, m	3.56, s	4.11, s
5	2.57, d (11.7)	3.50, m	3.17, m	2.57, d (11.7)	2.47, s
6	3.90, dd (11.7, 8.5)	3.72, t (10.2)	4.72, dd (10.4, 9.5)	3.90, dd (11.7, 8.5)	4.30, dd (11.5, 9.0)
7	3.33, m	3.26, m	3.06, m	3.31, td (11.2, 2.9)	3.52, m
8a	5.31, m	4.93, td (10.6, 2.1)	5.03, td (10.9, 4.0)	5.32, d (12.0)	1.88, dd (15.8, 7.6)
8b	—	—	—	—	2.64, m
9a	5.24, m	2.47, m	2.49, dt (6.8, 3.4)	5.29, s	1.45, m
9b	—	2.71, dd (13.4, 10.9)	1.59, dd (14.4)	—	2.35, ddt (11.5, 7.7)
13a	5.77, d (3.0)	5.65, d (2.9)	5.79, d (2.8)	5.77, d (2.7)	5.47, d (3.3)
13b	6.32, d (3.0)	6.22, d (2.9)	6.31, d (2.8)	6.33, d (2.7)	6.18, d (3.3)
14	1.92, m	2.47, m	1.56, s (14.4)	1.92, s	1.40, s
15	1.72, s	2.34, s	2.29, s	1.72, s	1.75, s
16	—	—	3.24, s	—	—
2′	2.62, m	2.43, d (5.5, 2.8)	2.41, m	2.42, dd (15.0, 7.5)	—
3′	1.22, d (7.0)	1.21, t (7.6)	1.19, t (7.6)	1.20, t (7.5)	—
4′	1.22, d (7.0)	—	—	—	—

a 600 MHz in CDCl_3_.

b 500 MHz in CDCl_3_.

**TABLE 3:** ^13^C NMR spectroscopic data for compounds **8–12**.

No.	1^a^	2^b^	3^a^	4^a^	5^a^	6^b^	7^a^	8^b^	9^b^	10^b^	11^b^	12^a^
	*δ*_C_	*δ*_C_	*δ*_C_	*δ*_C_	*δ*_C_	*δ*_C_	*δ*_C_	*δ*_C_	*δ*_C_	*δ*_C_	*δ*_C_	*δ*_C_
1	83.3	83.2	83.2	84.7	84.6	82.0	84.8	80.6	133.7	58.4	80.7	85.8
2	46.3	46.1	46.2	46.0	45.8	40.8	46.0	42.2	195.1	205.4	42.3	66.0
3	123.5	123.4	123.4	124.8	124.6	64.5	124.8	63.1	136.2	133.5	63.1	62.2
4	141.8	141.6	141.6	140.7	140.4	67.6	140.9	67.4	169.4	177	67.4	68.7
5	64.2	64.0	64.1	65.1	64.3	61.2	65.3	60.4	51.7	52.6	60.4	59.0
6	78.5	78.4	78.4	79.3	79.1	75.4	79.3	75.4	81.5	78.7	75.5	78.3
7	46.3	46.1	46.1	48.2	54.1	46.5	48.4	48.3	55.2	50.1	48.2	44.4
8	72.3	72.5	72.6	74.1	75.8	73.1	73.9	71.9	69.3	71.1	72.0	31.1
9	123.3	123.0	123.0	36.5	37.4	35.2	36.4	122.2	44.5	45.1	122.3	23.9
10	140.6	141.1	141.0	144.1	144.8	140.7	144.1	138.9	144.8	76.5	139.0	75.8
11	137.2	137.3	137.1	137.1	41.1	136.7	137.2	135.7	136.2	136.2	135.6	140.2
12	169.4	169.4	169.4	169.4	178.1	168.8	169.4	168.7	168.5	169.3	168.7	169.7
13	123.3	123.1	123.2	122.6	15.4	122.8	122.6	124.7	122.0	124.2	124.8	120.3
14	24.6	24.7	24.7	117.7	117.7	118.2	117.6	24.9	21.4	24.4	24.9	28.9
15	17.7	17.7	17.7	17.8	17.8	18.7	17.8	19.8	20.0	20.40	19.8	19.7
16	—	—	—	—	—	—	—	—	—	50.4	—	—
1′	167.1	176.0	172.5	172.4	167.0	176.4	167.0	176.3	173.3	173.6	173.7	—
2′	127.1	41.5	43.6	43.7	127.3	34.4	127.3	34.3	27.8	27.8	27.9	—
3′	140.8	26.6	25.8	25.8	140.1	18.9	140.3	19.2	9.1	9.1	9.1	—
4′	16.14	11.9	22.5	22.6	16.1	19.1	16.0	18.8	—	—	—	—
5′	20.7	16.9	22.6	22.6	20.7	—	20.7	—	—	—	—	—

a 125 MHz in CDCl_3_.

b 150 MHz in CDCl_3_.

#### 2.3.9 Artemvulactone P (**9**)

Colorless oil; [α]_D_^20^ + 74 (c 0.38, MeOH); UV (MeOH) *λ*_max_ (log *ε*) 257 (2.68) nm; IR (KBr) *ν*_max_/cm^−1^: 2,942, 1,773, 1,737, 1,688, 1,433, 1,255, 1,197, 1,137, 1,000. ^1^H and ^13^C NMR data (Tables 2, 3); HRESIMS *m/z* 317.1370 [M + H]^+^ (calcd for C_18_H_20_O_5_, 317.1384).

#### 2.3.10 Artemvulactone Q (**10**)

White powder; [α]_D_^20^ + 127 (c 0.18, MeOH); UV (MeOH) *λ*_max_ (log *ε*) 223 (4.33) nm; IR (KBr) *ν*_max_/cm^−1^: 2,944, 1,770, 1,736, 1,629, 1,460, 1,269, 1,183, 1,143, 1,022. ^1^H and ^13^C NMR data (Tables 2, 3); HRESIMS *m/z* 349.1644 [M + H]^+^ (calcd for C_19_H_24_O_6_, 349.1646).

#### 2.3.11 Artemvulactone R (**11**)

White powder; [α]_D_^20^ + 103 (c 0.23, MeOH); UV (MeOH) *λ*_max_ (log *ε*) 208 (4.38) nm; IR (KBr) *ν*_max_/cm^−1^: 3,465, 2,933, 1,761, 1,736, 1,373, 1,274, 1,241, 1,142, 999. ^1^H and ^13^C NMR data (Tables 2, 3); HRESIMS *m/z* 357.1301 [M + Na]^+^ (calcd for C_18_H_22_O_6_Na, 357.1309).

#### 2.3.12 Artemvulactone S (**12**)

White powder; [α]_D_^20^ + 131 (c 0.18, MeOH); UV (MeOH) *λ*_max_ (log *ε*) 211 (3.96) nm; IR (KBr) *ν*_max_/cm^−1^: 3,328, 2,942, 1,769, 1,642, 1,452, 1,390, 1,253, 1,143, 1,018, 936. ^1^H and ^13^C NMR data (Tables 2, 3); HRESIMS *m/z* 315.1008 [M + H]^+^ (calcd for C_15_H_19_ClO_5_, 315.0994).

### 2.4 X-ray crystallographic analyses

All crystals were obtained by recrystallization from MeOH. The X-ray diffraction data of compounds **6**, **8**, **10**, and **12** were collected on an Agilent SuperNova four-circle instrument by means of Cu Kα radiation. The structures were solved by direct methods and refined by the full-matrix least-squares process on F^2^ using the SHELXTL or the Olex2 software package. X-ray data can be obtained free from the Cambridge Crystallographic Data Centre *via* https://www.ccdc.cam.ac.uk/structures/.

#### 2.4.1 Crystal structure determination of compound **6**

Crystal data for C_19_H_24_O_6_ (M = 348.38 g/mol): orthorhombic, space group P2_1_2_1_2_1_ (no. 19), a = 8.6975 (2) Å, b = 9.7500 (2) Å, c = 20.3304 (5) Å, V = 1,724.03 (7) Å^3^, Z = 4, T = 170.00 (10) K, μ(Cu Kα) = 0.823 mm^−1^, Dcalc = 1.342 g/cm^3^, 9,848 reflections measured (8.698° ≤ 2Θ ≤ 147.768°), 3423 unique (R_int_ = 0.0325, R_sigma_ = 0.0320) which were used in all calculations. The final R_1_ was 0.0356 (I > 2σ (I)) and wR_2_ was 0.0914 (all data). The goodness of fit on F^2^ was 1.047. Flack parameter: −0.04 (9). CCDC 2164101.

#### 2.4.2 Crystal structure determination of compound **8**

Crystal data for C_19_H_24_O_6_ (M = 348.38 g/mol): orthorhombic, space group P2_1_2_1_2_1_ (no. 19), a = 9.34750 (10) Å, b = 10.61860 (10) Å, c = 17.3505 (2) Å, V = 1,722.16 (3) Å^3^, Z = 4, T = 179.99 (10) K, μ(Cu Kα) = 0.824 mm^−1^, Dcalc = 1.344 g/cm^3^, 18,414 reflections measured (9.766° ≤ 2Θ ≤ 147.578°), 3,462 unique (R_int_ = 0.0271, R_sigma_ = 0.0160) which were used in all calculations. The final R_1_ was 0.0293 (I > 2σ(I)) and wR_2_ was 0.0776 (all data). The goodness of fit on F^2^ was 1.090. Flack parameter: 0.01 (5). CCDC 2164105.

#### 2.4.3 Crystal structure determination of compound **10**

Crystal data for C_19_H_24_O_6_ (M = 348.38 g/mol): monoclinic, space group P2_1_ (no. 4), a = 8.4422 (4) Å, b = 7.1913 (4) Å, c = 15.2596 (6) Å, β = 99.791 (4)°, V = 912.92 (8) Å^3^, Z = 2, T = 169.99 (10) K, μ(Cu Kα) = 0.777 mm^−1^, Dcalc = 1.267 g/cm^3^, 10,067 reflections measured (5.878° ≤ 2Θ ≤ 148.026°), 3411 unique (R_int_ = 0.0440, R_sigma_ = 0.0498) which were used in all calculations. The final R_1_ was 0.0445 (I > 2σ(I)) and wR_2_ was 0.1126 (all data). The goodness of fit on F^2^ was 1.053. Flack parameter: −0.08 (15). CCDC 2164102.

#### 2.4.4 Crystal structure determination of compound **12**

Crystal data for C_15_H_19_ClO_5_ (M = 314.75 g/mol): orthorhombic, space group P2_1_2_1_2_1_ (no. 19), a = 5.80960 (10) Å, b = 14.6880 (2) Å, c = 16.6063 (2) Å, V = 1417.04 (4) Å^3^, Z = 4, T = 200.00 (10) K, μ(Cu Kα) = 2.575 mm^−1^, Dcalc = 1.475 g/cm^3^, 14,845 reflections measured (8.036° ≤ 2Θ ≤ 147.536°), 2,827 unique (R_int_ = 0.0403, R_sigma_ = 0.0232) which were used in all calculations. The final R_1_ was 0.0300 (I > 2σ(I)) and wR_2_ was 0.0760 (all data). The goodness of fit on F^2^ was 1.090. Flack parameter: 0.002 (7). CCDC 2164103.

### 2.5 ECD calculations

Conformational analyses for new compounds were carried out using MOE software with MMFF94s. The obtained stable conformers were optimized at the b3lyp/6-31+g(d) level in the gas phase and further subjected to ECD calculations at cam-b3lyp/6-31+g(d) level in the PCM model of methanol using the Gaussian 09 program. The ECD spectra were weighted according to the Boltzmann distributions.

### 2.6 Cytotoxicity

For the cytotoxicity assay, RAW 264.7 cells were grown in DMEM containing 10% FBS and cultured at 37°C. Then, the cells were seeded in a 96-well plate (1 × 10^4^ cells/well) before the cells were incubated for 24 h in various concentrations of compounds (3.125–200 μM). Cell viability was examined using a CCK8-kit. The CCK8 solution (5 µl) was added to each well and incubated for 1–2 h at 37°C. The OD value at 450 nm was quantified by Graphpad software, and the corresponding 50% cytotoxic concentration (CC_50_) of the compounds was subsequently obtained.

### 2.7 Measurement of NO production

The level of accumulated nitrite in the culture media reflected the level of NO using the classic Griess reagent (Beyotime, Jiangsu, China). The RAW264.7 cells were seeded in a 96-well plate with a 6 ×10^4^ cells/ml density for the indicated time. Then, the cells were pretreated for 1 h with test compounds (0.25–4 μM), followed by stimulation with LPS (1 μg/ml) for 18 h. Finally, 50 ul of the culture supernatant was mixed with an equal amount of the Griess reagent, and the optical densities at 570 nm were read using a microplate reader. The nitric oxide concentration of the samples was calculated according to the standard curve.

### 2.8 RNA isolation and quantitative real-time PCR

RAW264.7 cells were seeded in a six-well plate (10 × 10^5^ cells/well) for the indicated time. Cells were pretreated with compound **1** (0.5, 1, and 4 μM) for 1 h and then stimulated with LPS (1 ug/mL) for 18 h. Total cellular RNA was extracted with Trizol reagent (TIANGEN, Beijing, China). One microgram of RNA per sample was reverse-transcribed into cDNA using the PrimeScript RT Reagent Kit (TAKARA, Dalian, China). qPCR assays were performed by the CFX96 Touch Real-Time PCR Detection System (Bio-Rad). The following primers were used: iNOS (forward, 5′-AAA CCC CAG GTG CTA TTC CC-3′; reverse, 5′-TGG GTC CTC TGG TCA AAC TC-3′), COX-2 (forward, 5′-ATT CCA AAC CAG CAG ACT CAT A-3′; reverse, CTT GAG TTT GAA GTG GTA ACC G-3′), and GAPDH (forward, 5′- GTC ATT GAG AGC AAT GCC AG-3′; reverse, 5′-GTG TTC CTA CCC CCA ATG TG-3′). All relative gene expression levels were normalized to the internal reference (GAPDH).

### 2.9 Western blotting

RAW264.7 cells with the indicated treatment were harvested and treated in a RIPA lysis buffer (Beyotime, Shanghai, China) containing a protease and phosphatase inhibitor cocktail (Beyotime, Shanghai, China) to obtain the lysates. Then, the total proteins were measured using the bicinchoninic acid (BCA) protein assay kit (Beyotime, Shanghai, China) and regulated by a loading buffer and the RIPA lysis buffer. The samples were separated by sodium dodecyl sulfate polyacrylamide gel electrophoresis (SDS-PAGE) and then transferred to polyvinylidene difluoride membranes (Millipore), followed by blocking with 5% non-fat milk. The membranes were incubated with primary antibodies overnight at 4°C, followed by incubation with specific secondary antibodies. Finally, the protein bands were detected using the ImageJ software (Bio-Rad, Hercules, CA, United States).

### 2.10 Molecular docking

In order to explore the possibility of compounds **1**–**6** binding to the INOS target, Autodock vina and AutoDockTools-1.5.6 (Vina, 2010; Forli et al., 2016) were used to predict the free binding energy. Autodock vina used the Broyden-Fletcher-Goldfarb-Shanno (BFGS) 19 method to obtain the optimal conformation (Nguyen et al., 2020). First, the three-dimensional structure of INOS (PDB ID:3E6T) was obtained from the Protein Data Bank (http://www.rcsb.org) (Burley et al., 2021), whose resolution was 2.5 Å. The three-dimensional structure of compounds **1–6** was built and optimized by ChemDraw Ultra 8.0. Pymol molecular graphics software and AutodockTools-1.5.6 were used to remove ligand, dehydrate, hydrogenate, and charge the target (Liang, 2003; Seeliger and de Groot, 2010). The cubic grid box was calculated by AutoDockTools-1.5.6 positioned at the center of (122.65, 114.0, 36.63) with a spacing of 0.375 Å. All docking parameters were set to default values, but the modes and exhaustiveness were set to 10. The docking results were further analyzed and presented using pymol.

## 3 Results and discussion

### 3.1 Structural elucidation of new compounds **1–12**

**FIGURE 1:**
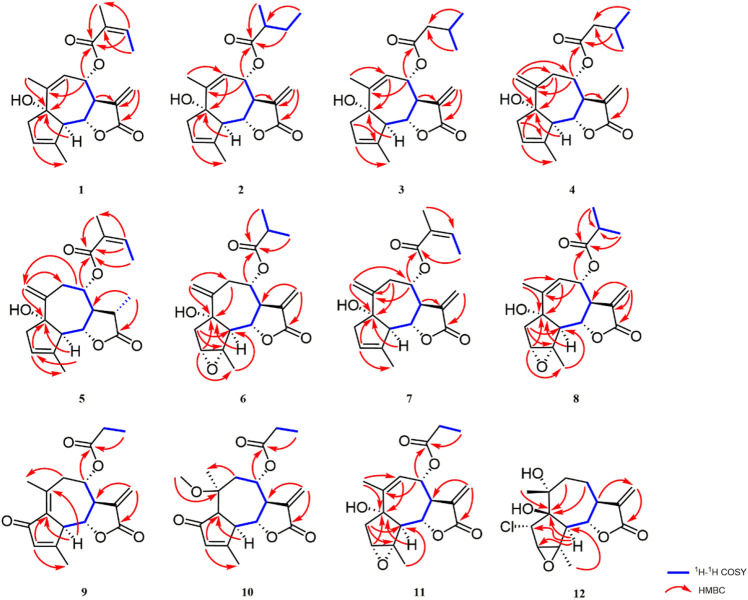
Key HMBC and ^1^H–^1^H COSY correlations of compounds **1**–**12**.

Artemvulactone H (**1**), obtained as a colorless oil, had a molecular formula of C_20_H_24_O_5_ deduced from the molecular ion at *m/z* 367.1504 ([M + Na]^+^, calcd for 367.1516) in the HRESIMS, indicating nine degrees of unsaturation. The IR absorptions of **1** suggested typical absorption bands for hydroxy (3,432 cm^−1^), carbonyl (1,767 and 1,711 cm^−1^), and olefinic (1,643 cm^−1^) functionalities. The ^1^H NMR spectrum (Table 1) of 1 displayed signals for a vinyl methyl observed at *δ*_H_ 1.94 (3H, m, H-15), a pair of terminal olefinic protons at *δ*_H_ 5.68 (1H, d, J = 3.0 Hz, H-13a), and 6.26 (1H, d, J = 3.0 Hz, H-13b), two oxygenated methines at *δ*_H_ 3.98 (1H, dd, 10.7, 9.2 Hz, H-6), and 5.42 (1H, ddd, 10.3, 3.6, 1.6 Hz, H-8). Apart from these dates, the ^13^C NMR (Table 3) and DEPT (Supplementary Figure S3) data of **1** with the aid of HSQC analysis exhibited 15 carbon resonances for 2 methyls, 2 methylenes, 6 methines, and 5 quarternary carbons. Comparison of the NMR spectra of 1 (Tables 1, 3) with 1α-hydroxy-3(4), 9(10), 11(13)-trien-8α-senecioyloxyguai-12,6α-olide showed that they had a closely relative stereochemistry except for a different ester side chain, indicating that **1** was a characteristic guaiane-type sesquiterpene lactone skeleton (Huang et al., 2010). A mixture of the ^1^H NMR spectrum at *δ*_H_ 6.21 (1H, qd, 7.3, 1.4 Hz, H-3′), 2.03 (3H, m, H-4′), and 1.94 (3H, m H-5′) and the ^13^C NMR spectrum at *δ*c 167.1 (C-1′), 127.1 (C-2′), 140.8 (C-3′), 16.14 (C-4′), and 20.7 (C-5′) of **1** displayed signals for an angeloyloxy group. The aforementioned conclusion was confirmed again by the correlation of H-3′/H_3_-4′ in the ^1^H–^1^H COSY spectrum and the correlations from H-3′ to C-1′, C-2′, and C-4′, from H_3_-4′ to C-2′, C-3′, and C-5′, and from H_3_-5′ to C-1′, C-2′, and C-3′ in the HMBC. The proton signal at *δ*_H_ 5.42 (H-8) disclosed a _3_J coupling with the carbonyl carbon signal at *δ*_C_ 167.1 (C-1′), indicating the connection of this group to C-8. The ^1^H–^1^H COSY data revealed two discrete proton spin systems, which were H-5/H-6/H-7/H-8, and H-3′/H_3_-4′. The HMBC correlations of H-6, H-7/C-12, H-5/C-6, and C-7 located the lactone group at C-6 and C-7 (Figure 1). Furthermore, the anguloid group and hydroxyl group were located at C-8 and C-1 based on the correlations of H-8 to C-1′, H_3_-14 to C-1, H-3 to C-1, and H-5 to C-1 in the HMBC experiment.

**FIGURE 2:**
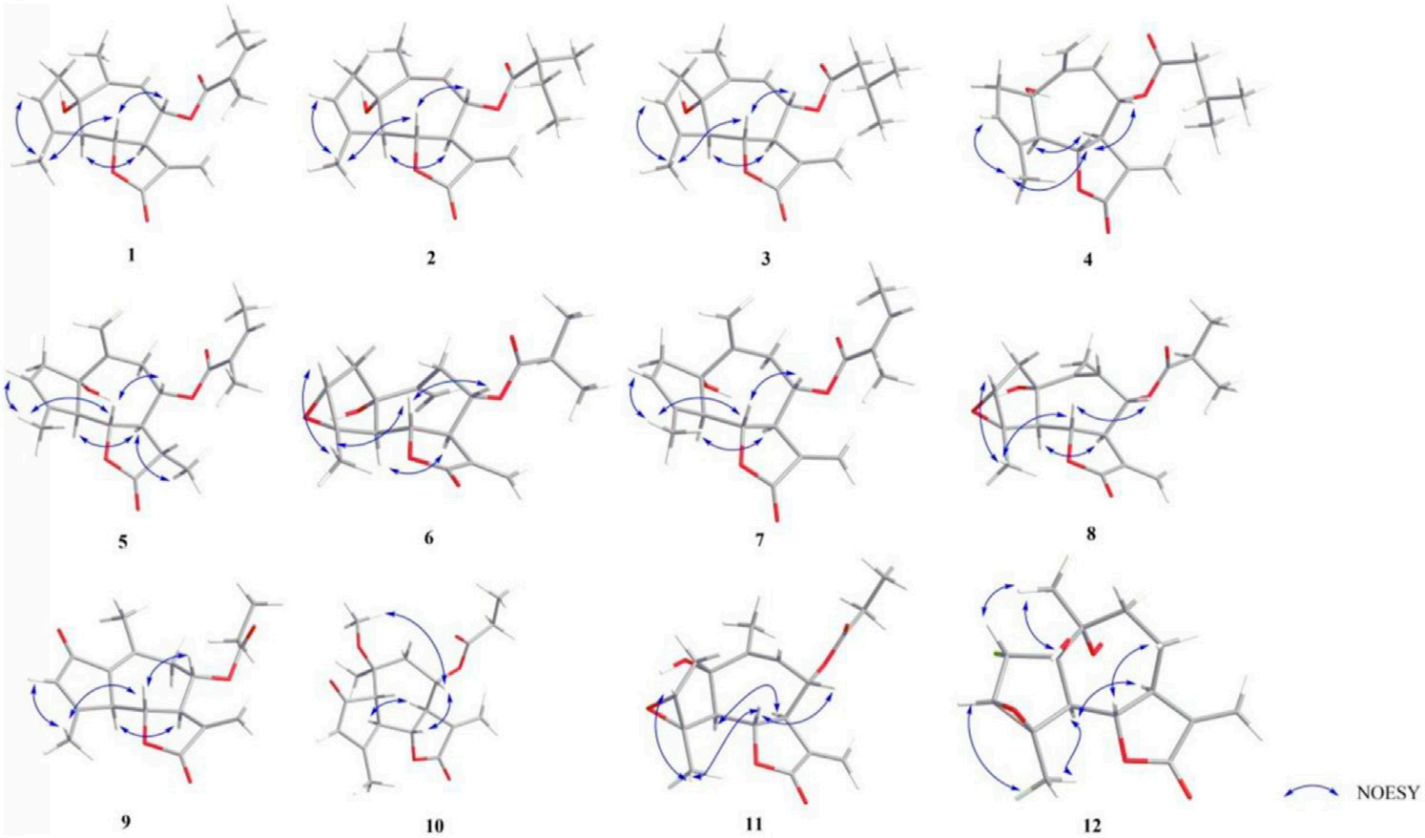
NOESY correlations of compounds **1**–**12**.

The relative configuration was determined by the correlations between H-7/H_2_-13, H-7/H-5, and H-6/H-8 in the NOESY spectrum (Figure 2). The absolute configuration of **1** was assigned according to the ECD calculations on the arbitrarily chosen enantiomers. Based on the evidence mentioned previously, the stereochemistry of **1** was eventually assigned as 1S,5R,6S,7R,8S.

Artemvulactone I (**2**) was obtained as a white amorphous powder. The molecular formula of **2** (C_20_H_26_O_5_) was established by the [M + Na]^+^ ion peak at *m/z* 369.1662 (calcd for 369.1672) in the HRESIMS (Supplementary Figure S18). The existence of hydroxy (3,465 cm^−1^), carbonyl (1,769 and 1,732 cm^−1^), and olefinic (1651 cm^−1^) groups were reflected in the IR spectrum (Supplementary Figure S20). The ^1^H/^13^C-NMR (Tables 1, 3), ^1^H–^1^H COSY, HMQC, and HMBC spectra of **2** were similar to **1**, with a difference in the C-8 substituent. The angeloyloxy group in **1** was replaced by the 2′-methylbutyryloxy unit in **2**. ^1^H NMR signals supported the aforementioned deduction at *δ*_H_ 2.43 (1H, m, H-2′), 1.50 (1H, m, H-3′a), 1.75 (1H, dt, 13.7, 7.4 Hz, H-3′b), 0.94 (3H, t, 7.4 Hz, H-4′), and 1.21 (3H, d, 7.0 Hz, H-5′) and the ^13^C NMR signals at *δ*_C_ 176.0 (C-1′), 41.5 (C-2′), 26.6 (C-3′), 11.9 (C-4′), and 16.9 (C-5′) of **2**. The absolute configuration of **2** was compared to **1**, which was determined by the NOESY and ECD spectra as shown.

**FIGURE 3:**
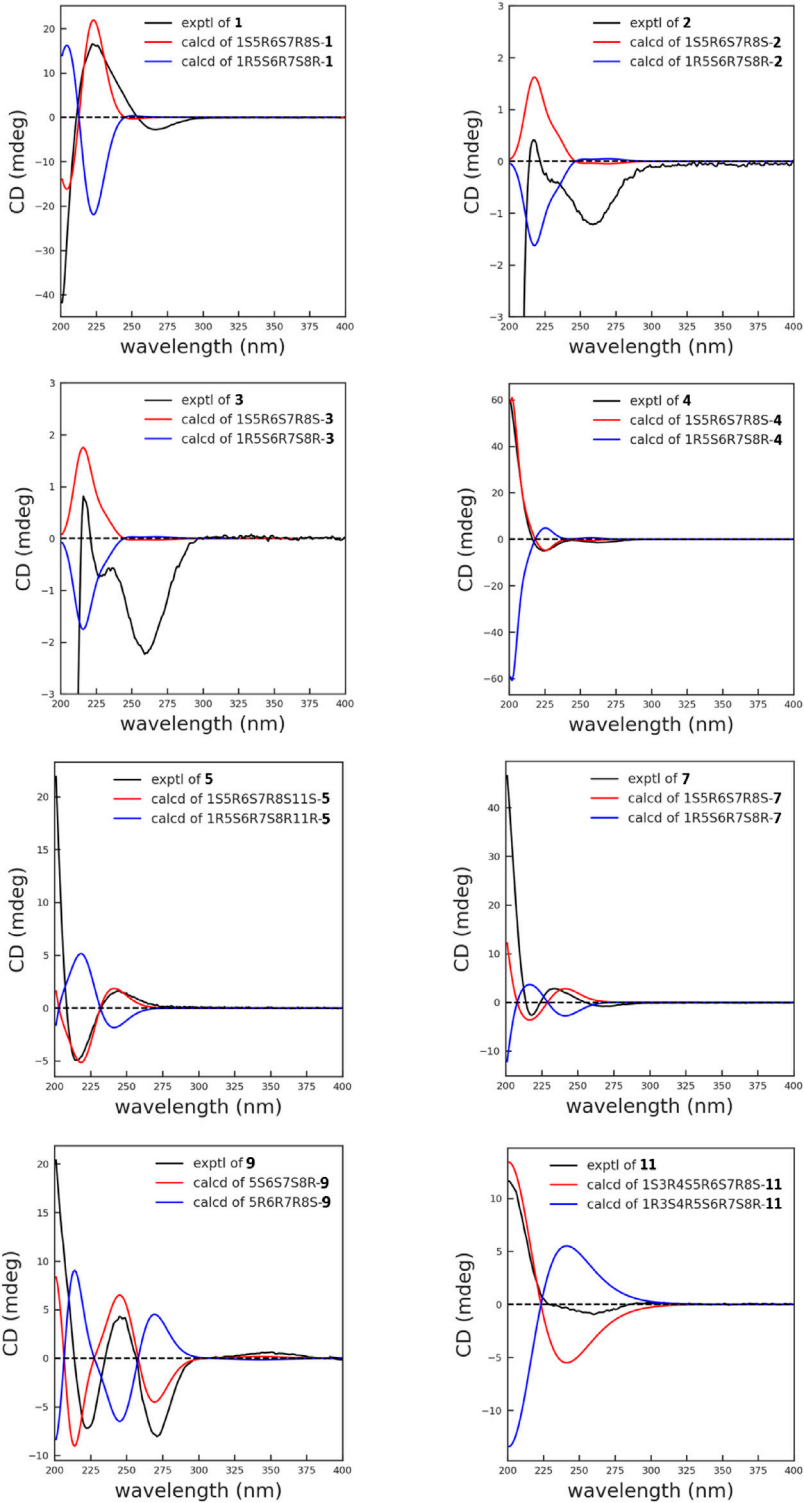
Experimental and calculated ECD spectra of compounds **1**–**5**, **7**, **9**, and **11** in MeOH.

Artemvulactone J (**3**) and Artemvulactone K (**4**) were both obtained as colorless oils and had the same molecular formula of C_20_H_26_O_6_ as that of **2** by HRESIMS. Similar to **2**, their NMR spectra revealed that **3** and **4** also possessed a guaianolide skeleton with characteristic α-methylene-γ-lactone [*δ*_H_ 5.69 (1H, d, 3.0 Hz, H-13a), 6.27(1H, d, 3.0 Hz, H-13b), *δ*_C_ 137.1 (C-11), 169.4 (C-12), 123.2(C-13)]. The ^1^H and ^13^C NMR spectra of **3** were almost superimposable to those of **2** except for the absence of 2′-methylbutyryloxy moiety. The ^1^H NMR signals at *δ*_H_ 2.27 (2H, m, H-2′), 2.15 (1H, m, H-3′), 0.99 (3H, d, 6.6, H-4′), and 0.99 (3H, d, 6.6, H-5′) indicated that **3** has an isovaleryloxy moiety rather than a 2′-methylbutyryloxy group, which was supported by the ^1^H–^1^H COSY cross peaks of H_2_-2′/H-3′/H_3_-4′(H_3_-5′) and HMBC spectrum from H-8 to C-1′ and H_2_-2′/H-3′ to C-1′. The 1D NMR spectra of **4** (Tables 1, 3) were very similar to compound **3**, except that a pair of terminal olefinic protons at *δ*_H_ 5.14 (1H, s, H-14a) and 5.40 (1H, s, H-14b) in **4**. The relative configurations of compounds **3** and **4** were determined by NOESY correlations and were similar to those of **2**. The 1S,5R,6S,7R,8S absolute configuration of compounds **3** and **4** (Figure 3) was verified based on comparing its ECD spectrum with that of **2**.

Artemvulactone L (**5**) was determined as C_20_H_26_O_5_ by HRESIMS ([M + Na]^+^, *m/z* 369.1660, calcd for 369.1672). The IR spectrum revealed the presence of hydroxy (3,469 cm^−1^), carbonyl (1,773 and 1,712 cm^−1^), and olefinic (1640 cm^−1^) groups. The ^1^H and ^13^C NMR spectra of **5** (Tables 1, 3) were comparable to **1** except for the presence of a pair of terminal olefinic protons at *δ*_H_ 5.21(1H, s, H-14a), *δ*_H_ 5.31(1H, s, H-14b), and the absence of a terminal double bond at C-13. The HMBC correlation between the proton signal at *δ*_H_ 4.97 (1H, td, 9.1, 4.6 Hz, H-8) and the carbonyl carbon signal at *δ*_C_ 167.0 (C-1′) demonstrated an angeloyl group attached to C-8 (Figure 1). Finally, the stereochemistry of **5** was determined in a manner similar to that of **1**.

**FIGURE 4:**
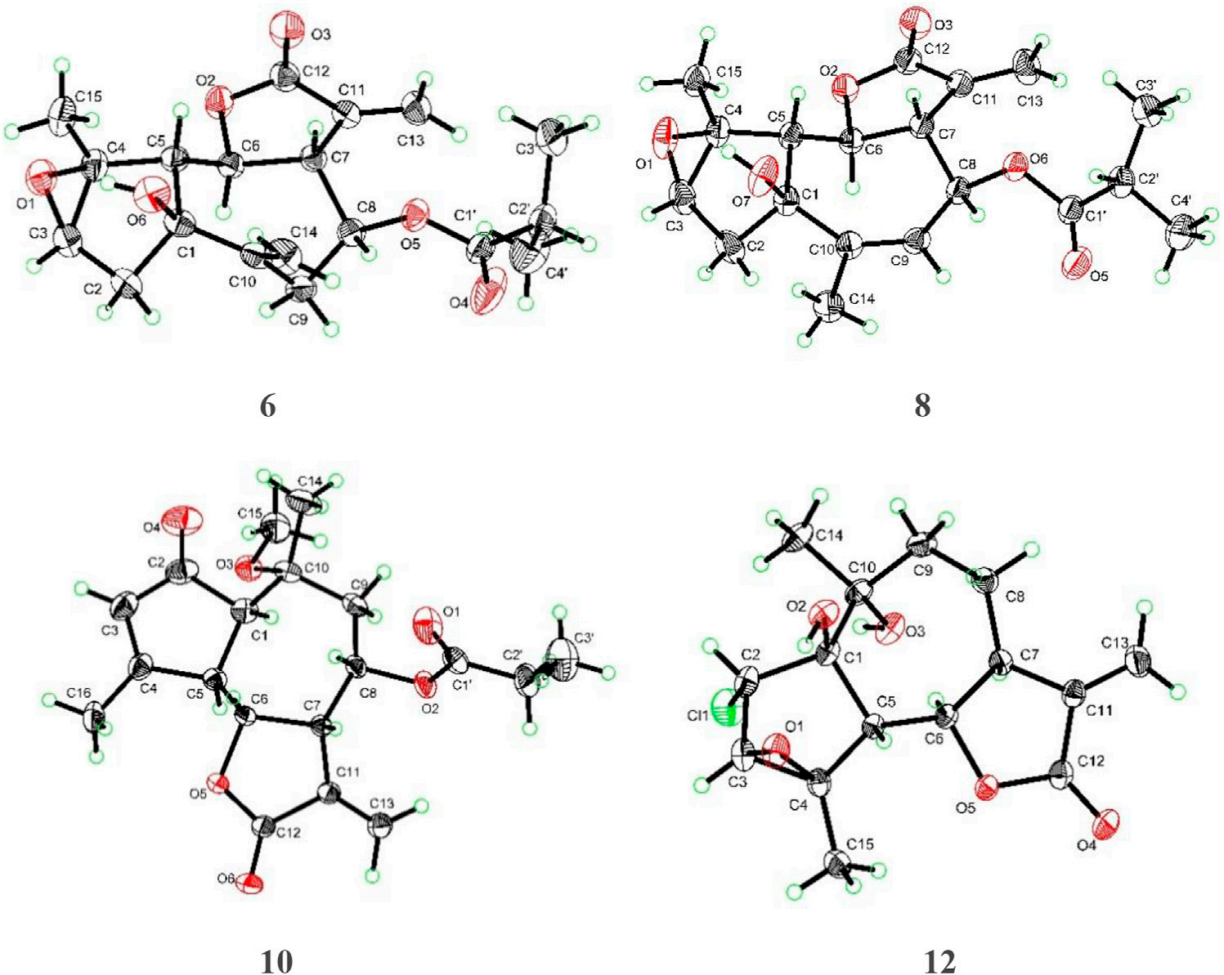
X-ray crystallographic analysis of compounds **6**, **8**, **10**, and **12**.

Artemvulactone M (**6**) was determined as C_19_H_24_O_6_ according to the HRESIMS ([M + Na]^+^, *m/z* 371.1450, calcd for 371.1465). The IR absorption bands at 3,495, 1,772, and 1,731 cm^−1^ were marks of hydroxy and carbonyl groups. The ^1^H and ^13^C NMR spectra of **6** were comparable to **4**, but the isovaleryloxy group is absent. It could be aided by the HMBC correlation between the proton signal at *δ*_H_ 4.96 (1H, td, 9.9, 2.1 Hz, H-8) and the carbonyl carbon signal at *δ*_C_ 176.4 (C-1′). In addition, the main difference was ascribed to the absence of a cyclic olefinic bond at C-3 and C-4 in **6**, but an additional ring connected via an oxygen atom. The aforementioned reasoning was based on the correlation of H-2′/H_3_-3′ (H_3_-4′) in the ^1^H−^1^H COSY spectrum and the cross peak from H-2′/H_3_-3′ (H_3_-4′) to C-1′ in the HMBC spectrum (Figure 1). The single-crystal X-ray diffraction experiment with Cu Kα radiation [CCDC,2164101] confirmed the 2D structure of **6** and defined its absolute configuration as 1S,3R,4S,5R,6S,7R,8S. Therefore, the absolute configuration of compound **6** was established as shown (Figure 4).

Artemvulactone N (**7**) was obtained as a colorless oil. The molecular formula C_20_H_24_O_5_ was deduced from the HRESIMS at *m/z* 345.1703 [M + H]^+^ (calcd for 345.1697). The IR spectra revealed that **7** possessed hydroxy, carbonyl, and olefinic groups. In the NMR spectra of **7** (Tables 1, 3), the signals at *δ*_H_ 5.16 (1H, s, H-14a), 5.44 (1H, s, H-14b), *δ*_C_ 144.1 (C-10), and 117.6 (C-14) revealed that a terminal double bond was located at C-10, which was supported by the HMBC correlations from H_2_-14 to C-1 and C-8. The NMR spectra of **7** (Tables 1, 3) were very similar to compound **4**, except that the isovaleryloxy substituent at C-8 in **4** was replaced by an angeloyloxy group in **7**. The absolute configuration of 1S,5R,6S,7R,8S was confirmed by the comparison of experimental and calculated ECD spectra (Figure 3).

Artemvulactone O (**8**) gave a molecular formula of C_19_H_24_O_6_ as defined by the HRESIMS ion at *m/z* 371.1456 [M + Na]^+^ (calcd for 371.1465). The presence of hydroxy (3,476 cm^−1^), carbonyl (1,769 and 1,730 cm^−1^), and olefinic (1,658 cm^−1^) functionalities was evident from the spectroscopic data. The ^1^H and ^13^C NMR spectra of **8** manifested that it was structurally similar to **6**. The main difference was the position of a double bond, which was verified by the HMBC correlations from H_3_-14 to C-9 (Figure 1). The absolute configuration of 1S,3R,4S,5R,6S,7R,8S was determined by means of a single crystal X-ray crystallographic diffraction experiment with Cu Kα radiation (CCDC, 2164105, Figure 4).

Artemvulactone P (**9**) was isolated as a colorless oil. According to the HRESIMS ([M + H]^+^, *m/z* 317.1370, calcd for 317.1384) and the 1D NMR spectrum, the molecular formula of **9** was assigned as C_18_H_20_O_5_. The IR absorption bands at 1,773, 1,737, and 1,673 cm^−1^ confirmed the existence of carbonyl and olefinic groups, with one less hydroxyl group than compound **1**. Additionally, the propionyl group was attached to oxygen-linked carbon at C-8, which could be demonstrated by the signals at *δ*_C_ 173.3 (C-1′), 27.8 (C-2′), and 9.1 (C-3′). In the 1D NMR spectrum, the characteristic signals of methylene [*δ*_H_ 2.65 (2H, m, H-2); *δ*_C_ 46.3 (C-2)] in **1** were replaced by a carbonyl in **9** at C-2 (*δ*_C_ 195.1). Based on the application of the TD-SCF ECD calculation method, the similarity of the calculated ECD spectrum with its experimental spectrum indicated the 5R,6R,7S,8R configuration of **9** (Figure 3).

Artemvulactone Q (**10**) showed the same molecular formula as that of compound **8**. The carbonyl and olefinic groups also existed in the structure of **10**, which were attributable to the IR absorptions at 1,770, 1,736, and 1,629 cm^−1^. The compound **10** differed from **9** by one more methoxy group and one less cyclic olefinic bond in its chemical structure at C-10. The aforementioned analysis was confirmed by the ^1^H–^1^H COSY correlations of H-1/H-9/H_3_-14/H_3_-16 and HMBC correlations from *δ*_H_ 3.24 (–OCH_3_) to *δ*_C_ 76.5 (C-10). The absolute configuration of compound **10** was established based on a single-crystal X-ray crystallographic diffraction experiment with Cu Kα radiation (Figure 4).

Artemvulactone R (**11**) was obtained as a white powder and assigned a molecular formula of C_18_H_22_O_6_ by HRESIMS and ^13^C NMR data. The IR absorption bands of **11** implied hydroxy, carbonyl, and olefinic groups. The 1D NMR data (Tables 2, 3) revealed a definite structural variation between **11** and **8**. The main difference was that, compound **11** possessed one less methyl group at C-2′ [*δ*_H_ 2.42 (2H, dd, 15.0, 7.5 Hz, H-2′); *δ*_C_ 27.9 (C-2′)], which was defined based on the ^1^H−^1^H COSY correlations of H_2_-2′/H_3_-3′and HMBC correlations from H_3_-3′ to C-1′ and C-2′ (Figure 1). The calculated ECD spectrum for the absolute configuration was consistent with the experimental ECD spectrum of compound **11**.

Artemvulactone S (**12**), isolated as a white powder, had the molecular formula of C_15_H_19_ClO_5_, implying six degrees of unsaturation. The IR absorption bands at 3,328, 1,769, and 1,642 cm^−1^ suggested hydroxy, carbonyl, and olefinic groups. The resonances for two methyl singlets at *δ*_Η_ 1.40 (H_3_-14) and 1.75 (H_3_-15), a pair of terminal olefinic protons at *δ*_Η_ 5.47 (1H, d, J = 3.3 Hz, H-13a) and 6.18 (1H, d, J = 3.3 Hz, H-13b) were observed in the ^1^H NMR spectrum. The ^13^C NMR and HSQC spectra revealed that **12** is a sesquiterpene lactone. And the absolute configuration of compound **12** was determined by single crystal X-ray diffraction (Cu Kα) (Figure 4).

**FIGURE 5:**
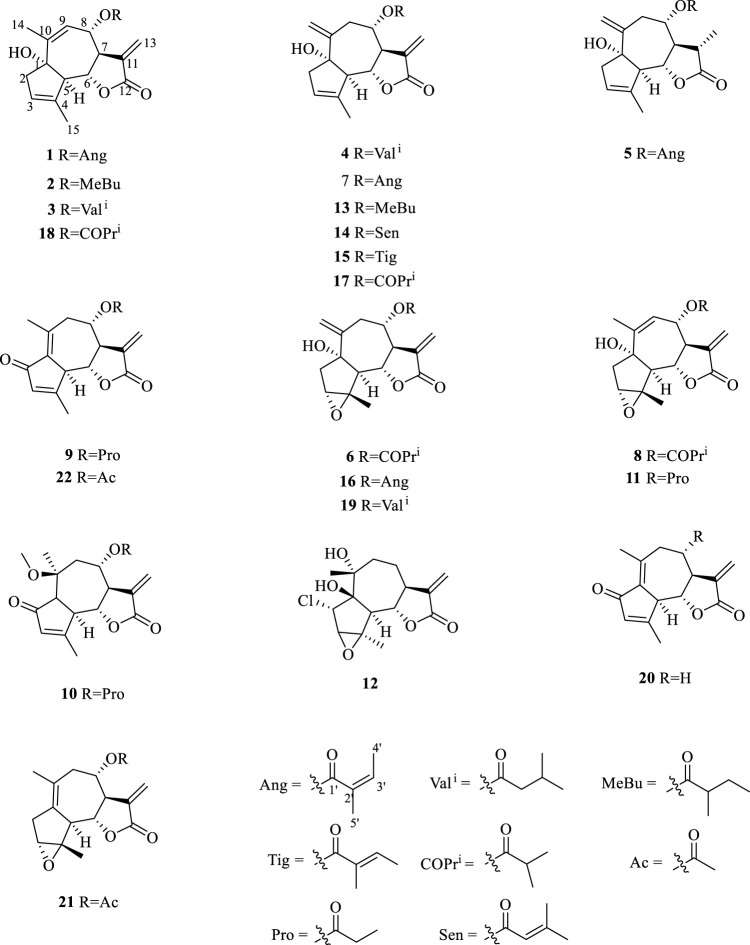
Structures of compounds **1**–**22** isolated from *A. vulgaris*.

Additionally, 10 known compounds were isolated and their structures were identified by comparing their physical and spectroscopic data with the reported data (Figure 5). The known compounds were identified as 1α-hydroxy-8α-methylbutyroxy-3(4),10(14),11(13)-trienguai-12,6α-olide (**13**) (Huang et al., 2010), 1α-hydroxy-8α-senecioyloxy-3(4),10(14),11(13)-trienguai-12,6α-olide (**14**) (Huang et al., 2010), 8-epi-Tiglylrupicolin B (**15**) (Ober et al., 1985), 8-angeloyloxy-1α-hydroxy-3α, 4α-epoxy-5α, 7αH-10(14), 11(13)-guaiadien-12,6α-olide (**16**) (Jin et al., 2004), 8-epi-isobutyrylrupicolin B (**17**) (Ober et al., 1984), 8-epi-isobutyrylrupicolin (**18**) (Ober et al., 1984), 3α,4α-epoxyrupicolin E (**19**) (Jin et al., 2004), dehydroleucodine (**20**) (de Heluani et al., 1989), (+)-Arteglasin A (**21**) (Lee et al., 1971), and Dehydromatricarin (**22**) (Bohlmann and Zdero, 1978).

### 3.2 Anti-inflammatory effects for the intervention of NO production in LPS-induced RAW264.7 cells

Inhibition of NO overexpression is an essential indicator for evaluating small molecules with anti-inflammatory activities. Thus, the inhibitory effects of compounds **1**–**22** on LPS-mediated NO production were tested, and it was found that isolated sesquiterpenoids except compound **5** exhibited a concentration-dependent NO production inhibitory activity with IC_50_ values ranging from 1.0 to 3.6 μM (Table 4). It was preliminarily concluded that the isolated compounds had the potential to be developed into anti-inflammatory drugs.

**TABLE 4:** Cytotoxicity against RAW264.7 cells and NO Inhibition of **1–12** and Dexamethasone toward LPS-Induced RAW264.7 cells (Mean ± SD).

Compound	IC_50_ (μM)	CC_50_ (μM)	Compound	IC_50_ (μM)	CC_50_ (μM)
**1**	1.1 ± 0.1	>10	**13**	1.5 ± 0.1	>15
**2**	1.2 ± 0.3	>10	**14**	1.4 ± 0.2	>15
**3**	2.8 ± 0.1	>10	**15**	1.2 ± 0.1	>10
**4**	3.6 ± 0.1	>20	**16**	1.1 ± 0.2	>10
**5**	>10	>100	**17**	1.5 ± 0.1	>20
**6**	3.1 ± 0.1	>20	**18**	1.1 ± 0.1	>20
**7**	2.1 ± 0.6	>20	**19**	1.0 ± 0.2	>25
**8**	3.2 ± 0.1	>20	**20**	1.8 ± 0.1	>70
**9**	1.9 ± 0.8	>20	**21**	1.2 ± 0.2	>20
**10**	2.1 ± 0.1	>10	**22**	1.8 ± 0.2	>10
**11**	1.9 ± 0.4	>15	Dexamethasone	4.3 ± 0.3	—
**12**	2.7 ± 0.5	>40			

**FIGURE 6:**
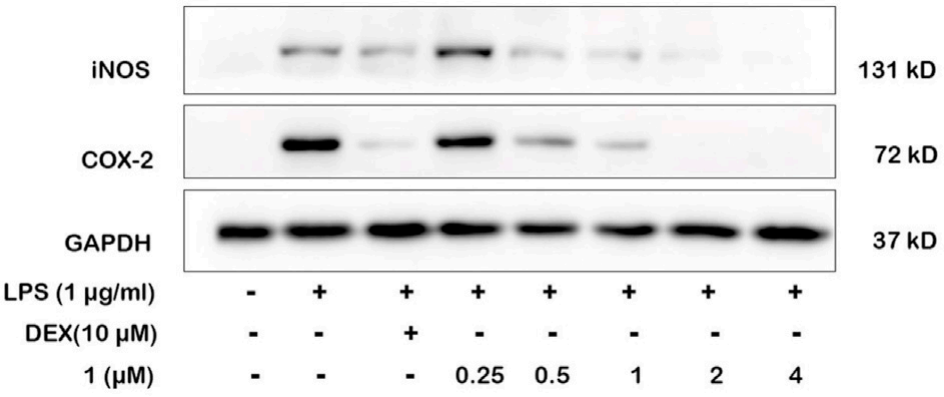
Effects of **1** and positive control on iNOS and COX-2 protein levels in RAW264.7 cells by Western blotting. Cells were pretreated with different concentrations of **1** (0.25, 0.5, 1, 2, and 4 μM) and DEX (10 μM) for 1 h and then stimulated with LPS (1 μg/ml) for 24 h.

**FIGURE 7:**
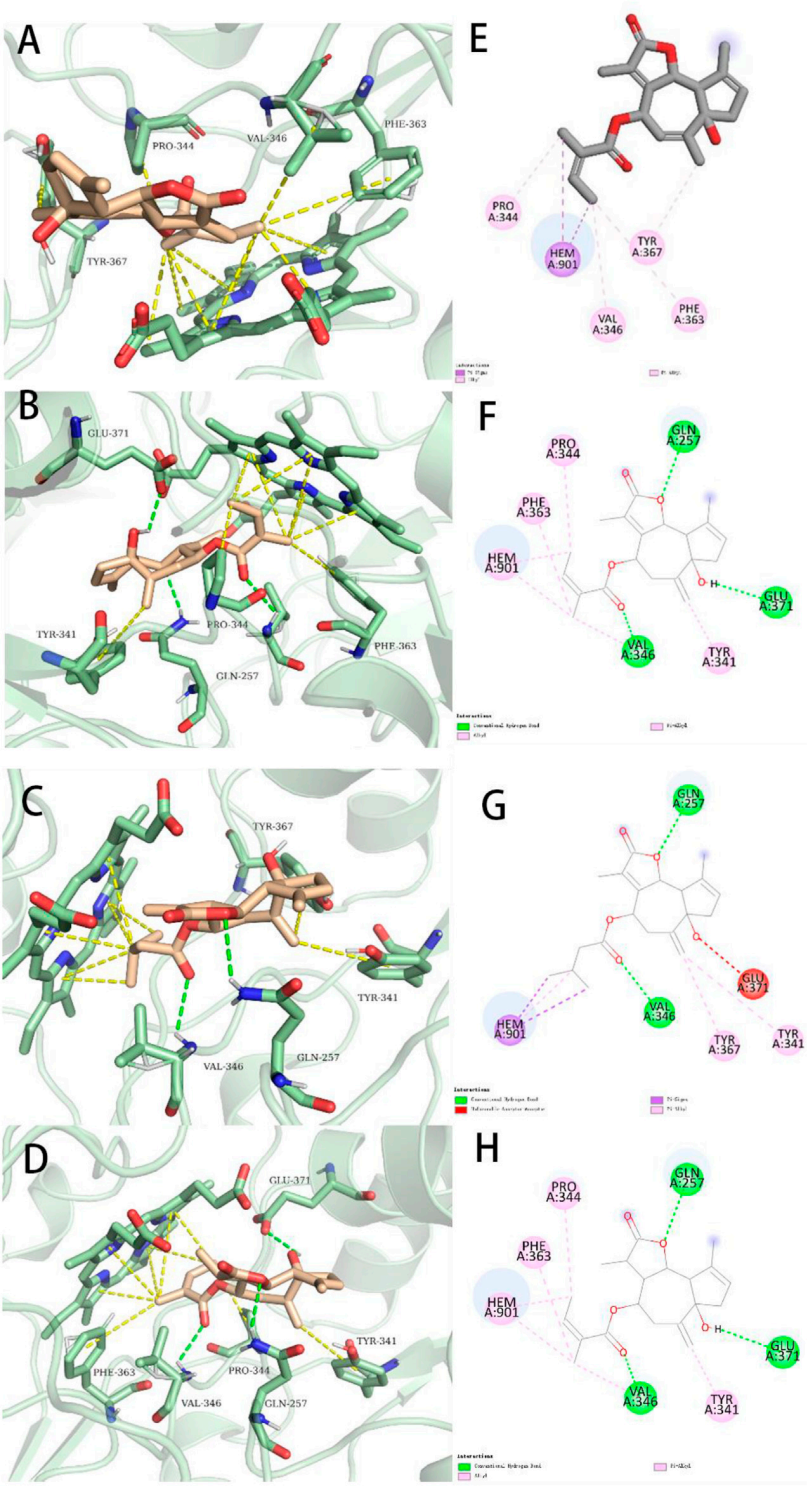
Representations of lowest energy docking poses of compounds **1 (A)**, **7 (B)**, **4 (C)**, and **5 (D)** bound to the iNOS protein (PDB ID:3E6T). Intermolecular interactions between iNOS and compounds **1 (E)**, **7 (F)**, **4 (G)**, and **5 (H)** are highlighted by 2-D interaction maps.

NO production is directly related to the expressions of iNOS and COX-2. We selected the most active compound **1** for gene and protein level verification. The results of experiments demonstrated that compound **1** at 4 μM reduced more expressions of iNOS and COX-2 than the positive drug (Dexamethasone) in LPS-induced macrophages by quantitative real-time PCR and Western blotting (Figures 6, 7). By comparing the structure and activity of the compounds, we preliminarily summarize the structure–activity relationship of guaiane-type sesquiterpene lactones in inhibiting inflammation: α-methylene-γ-lactone, α,β-unsaturated carbonyl moieties, and the ester side chain at C-8 are critical for cytotoxic activity.

**FIGURE 8:**
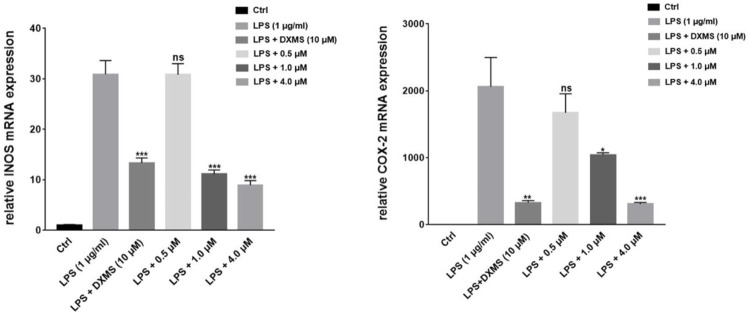
Compound **1** on the downregulation of LPS-induced iNOS and COX-2 mRNA expression in RAW264.7 cells. *p < 0.05, **p < 0.01, ***p < 0.001, compared to the LPS-treated groups.

To better compare the binding ability of docking compounds, dexamethasone and INOS were used for redocking. Molecular docking results showed that **1**–**12** and dexamethasone had a good interaction with the INOS targets by targeting residues in pockets. Figure 8 revealed the predicted geometry of 1, 7, 4 and 5 bound to the INOS protein. It was conformable to the results obtained through biological experiments. The free-binding energy and the number of binding residues are shown in Table 5, and that compounds **7**, **5**, and **4** were predicted to possess a stronger association with the protein. Previous research has shown that the type of substituent at C-8 also affects the compound activity: isovaleryloxy > acetyl > angeloyloxy > methylbutyryloxy (Sun et al., 2020). Combined with biological experiments, the hydroxyl group at C-1 was inferred to influence compound activity, and the following trend in activity for the side chain was summarized: angeloyloxy > methylbutyryloxy > isovaleryloxy. The aforementioned information rationalizes the results of molecular docking and biological experiments.

**TABLE 5:** Binding energy, Hydrophobic and Hydrogen bonds formed between INOS (PDB ID:3E6T) and ligands (**1–12** and INOS co-crystal ligand 2650707-81-4).

Compound	Binding energy (kcal/mol)	Hydrophobic and Hydrogen bonds
**7**	−10.2	HEM-901, PHE-363, PRO-344, GLN-257, GLU-371, TYR-341, VAL-346
**5**	−9.8	HEM-901, PHE-363, PRO-344, GLN-257, GLU-371, TYR-341, VAL-346
**4**	−9.5	HEM-901, GLN-257, VAL-346, TYR-367, TYR-341
**2**	−9.3	HEM-901, GLN-257, VAL-346, TYR-367
**3**	−9.3	HEM-901, GLN-257, VAL-346, TYR-367
**6**	−9.1	TYR-367, HEM-901, VAL-346, PRO-344, GLN-257
**1**	−8.9	HEM-901, PRO-344, VAL-346, ASP-376, ARG-362
**8**	−8.9	ARG-382, ASP-376, VAL-346, TYR-367, HEM-901
**11**	−8.3	HEM-901, PRO-344, VAL-346, ASP-376, ARG-382
**9**	−8.2	HEM-901, GLN-257, VAL-346, ARG-260, PRO-344, TYR-367, TYR-341
**12**	−8.1	TRP-457, GLNN-257, GLU-371, ARG-375
**10**	−7.6	ARG-260, ARG-375, TYR-367
Dexamethasone	−8.8	ARG-382, ALA-276, HEM-901, TYR-485, TRP-457

## 4 Conclusion

In conclusion, in our endeavor to search for anti-inflammatory compounds from *Artemisia vulgaris* L., 12 new and 10 known guaiane-type sesquiterpenoids were isolated and identified, of which compound **1** was the most potent inhibitor on NO release in LPS-stimulated RAW264.7 cells. The biological data confirmed that the expression of inflammatory enzymes of iNOS and COX-2 was suppressed by **1** in LPS-stimulated RAW264.7 cells. The binding interactions of compound **1** with iNOS also confirmed the conclusion. Further mechanisms need to be further explored. Accordingly, **1** displayed the therapeutic potential for modulating inflammation.

## Data Availability

The datasets presented in this study can be found in online repositories. The names of the repository/repositories and accession number(s) can be found in the article/Supplementary Material.
