# Supplementary material for: Sesquiterpene lactones from Artemisia vulgaris L. as potential NO inhibitors in LPS-induced RAW264.7 macrophage cells
Source: Front Chem. 2022 Sep 1;10:948714. doi: 10.3389/fchem.2022.948714 (PMC9475137; doi:10.3389/fchem.2022.948714)
Supplement: Supplementary file 1 [file DataSheet1.docx]

Sesquiterpene lactones from *Artemisia vulgaris L*. as potential NO inhibitors in LPS-induced RAW264.7 macrophage cells

Xiang-yu Chen ^a,b,c,d,1^, Tao Liu ^a,b,c,d,1^, Yu-ze Hu ^f^, Tian-tian Qiao ^a,b,c,d^,

Xiu-juan Wu ^a,b,c,d^, Ping-hua Sun ^f^, Chui-wen Qian ^a,b,c,d,e^, Zhe Ren ^a,b,c,d,e,*^,

Jun-xia Zheng ^g,*^, Yi-fei Wang ^a,b,c,d,e,*^

^a^ Department of Cell Biology, College of Life Science and Technology, Jinan University

^b^ Guangdong Province Key Laboratory of Bioengineering Medicine

^c^ Guangdong Provincial biotechnology drug & Engineering Technology Research Center

^d^ National Engineering Research Center of Genetic Medicine

^e^ GuangZhou (Jinan) Biomedical Research and Development Center Co. Ltd, Guangzhou, China.

^f^ College of Pharmacy, Jinan University, Guangzhou 510632, PR China

^g^ School of Biomedical and Pharmaceutical Sciences, Guangdong University of Technology, Guangzhou 510006, PR China

**Contents of Supporting Information**

目录

[**Table S1**. Crystal data and structure refinement for Compound **6**	7](#_Toc102205807)

[**Table S1**. Crystal data and structure refinement for Compound **8**	8](#_Toc102205808)

[**Table S3**. Crystal data and structure refinement for Compound **10**	9](#_Toc102205809)

[**Table S4** Crystal data and structure refinement for Compound **12**	10](#_Toc102205810)

[**Figure S1**. ^1^H NMR Spectrum of Compound **1** in CDCl_3_	11](#_Toc102205811)

[**Figure S2**. ^13^C NMR Spectrum of Compound **1** in CDCl_3_	11](#_Toc102205812)

[**Figure S3**. DEPT 135 Spectrum of Compound **1** in CDCl_3_	12](#_Toc102205813)

[**Figure S4**. ^1^H–^1^H COSY Spectrum of Compound **1** in CDCl_3_	12](#_Toc102205814)

[**Figure S5**. HSQC Spectrum of Compound **1** in CDCl_3_	13](#_Toc102205815)

[**Figure S6**. HMBC Spectrum of Compound **1** in CDCl_3_	13](#_Toc102205816)

[**Figure S7**. NOESY spectrum of compound **1** in CDCl_3_	14](#_Toc102205817)

[**Figure S8**. (+) HRESIMS Spectrum of Compound **1**	14](#_Toc102205818)

[**Figure S9**. UV Spectrum of Compound **1**	15](#_Toc102205819)

[**Figure S10**. IR (KBr disc) Spectrum of Compound **1**	15](#_Toc102205820)

[**Figure S11**. ^1^H NMR Spectrum of Compound **2** in CDCl_3_	16](#_Toc102205821)

[**Figure S12**. ^13^C NMR Spectrum of Compound **2** in CDCl_3_	16](#_Toc102205822)

[**Figure S13**. DEPT 135 Spectrum of Compound **2** in CDCl_3_	17](#_Toc102205823)

[**Figure S14**. ^1^H–^1^H COSY Spectrum of Compound **2** in CDCl_3_	17](#_Toc102205824)

[**Figure S15**. HSQC Spectrum of Compound **2** in CDCl_3_	18](#_Toc102205825)

[**Figure S16**. HMBC Spectrum of Compound **2** in CDCl_3_	18](#_Toc102205826)

[**Figure S17**. NOESY spectrum of compound **2** in CDCl_3_	19](#_Toc102205827)

[**Figure S18**. (+) HRESIMS Spectrum of Compound **2**	19](#_Toc102205828)

[**Figure S19**. UV Spectrum of Compound **2**	20](#_Toc102205829)

[**Figure S20**. IR (KBr disc) Spectrum of Compound **2**	20](#_Toc102205830)

[**Figure S21**. ^1^H NMR Spectrum of Compound **3** in CDCl_3_	21](#_Toc102205831)

[**Figure S22**. ^13^C NMR Spectrum of Compound **3** in CDCl_3_	21](#_Toc102205832)

[**Figure S23**. DEPT 135 Spectrum of Compound **3** in CDCl_3_	22](#_Toc102205833)

[**Figure S24**. ^1^H–^1^H COSY Spectrum of Compound **3** in CDCl_3_	22](#_Toc102205834)

[**Figure S25**. HSQC Spectrum of Compound **3** in CDCl_3_	23](#_Toc102205835)

[**Figure S26**. HMBC Spectrum of Compound **3** in CDCl_3_	23](#_Toc102205836)

[**Figure S27**. NOESY spectrum of compound **3** in CDCl_3_	24](#_Toc102205837)

[**Figure S28**. (+) HRESIMS Spectrum of Compound **3**	24](#_Toc102205838)

[**Figure S29**. UV Spectrum of Compound **3**	25](#_Toc102205839)

[**Figure S30**. IR (KBr disc) Spectrum of Compound **3**	25](#_Toc102205840)

[**Figure S31**. ^1^H NMR Spectrum of Compound **4** in CDCl_3_	26](#_Toc102205841)

[**Figure S32**. ^13^C NMR Spectrum of Compound **4** in CDCl_3_	26](#_Toc102205842)

[**Figure S33**. DEPT 135 Spectrum of Compound **4** in CDCl_3_	27](#_Toc102205843)

[**Figure S34**. ^1^H–^1^H COSY Spectrum of Compound **4** in CDCl_3_	27](#_Toc102205844)

[**Figure S35**. HSQC Spectrum of Compound **4** in CDCl_3_	28](#_Toc102205845)

[**Figure S36**. HMBC Spectrum of Compound **4** in CDCl_3_	28](#_Toc102205846)

[**Figure S37**. NOESY spectrum of compound **4** in CDCl_3_	29](#_Toc102205847)

[**Figure S38**. (+) HRESIMS Spectrum of Compound **4**	29](#_Toc102205848)

[**Figure S39**. UV Spectrum of Compound **4**	30](#_Toc102205849)

[**Figure S40**. IR (KBr disc) Spectrum of Compound **4**	30](#_Toc102205850)

[**Figure S41**. ^1^H NMR Spectrum of Compound **5** in CDCl_3_	31](#_Toc102205851)

[**Figure S42**. ^13^C NMR Spectrum of Compound **5** in CDCl_3_	31](#_Toc102205852)

[**Figure S43**. DEPT 135 Spectrum of Compound **5** in CDCl_3_	32](#_Toc102205853)

[**Figure S44**. ^1^H–^1^H COSY Spectrum of Compound **5** in CDCl_3_	32](#_Toc102205854)

[**Figure S45**. HSQC Spectrum of Compound **5** in CDCl_3_	33](#_Toc102205855)

[**Figure S46**. HMBC Spectrum of Compound **5** in CDCl_3_	33](#_Toc102205856)

[**Figure S47**. NOESY spectrum of compound **5** in CDCl_3_	34](#_Toc102205857)

[**Figure S48**. (+) HRESIMS Spectrum of Compound **5**	34](#_Toc102205858)

[**Figure S49**. UV Spectrum of Compound **5**	35](#_Toc102205859)

[**Figure S50**. IR (KBr disc) Spectrum of Compound **5**	35](#_Toc102205860)

[**Figure S51**. ^1^H NMR Spectrum of Compound **6** in CDCl_3_	36](#_Toc102205861)

[**Figure S52**. ^13^C NMR Spectrum of Compound **6** in CDCl_3_	36](#_Toc102205862)

[**Figure S53**. DEPT 135 Spectrum of Compound **6** in CDCl_3_	37](#_Toc102205863)

[**Figure S54**. ^1^H–^1^H COSY Spectrum of Compound **6** in CDCl_3_	37](#_Toc102205864)

[**Figure S55**. HSQC Spectrum of Compound **6** in CDCl_3_	38](#_Toc102205865)

[**Figure S56**. HMBC Spectrum of Compound **6** in CDCl_3_	38](#_Toc102205866)

[**Figure S57**. NOESY spectrum of compound **6** in CDCl_3_	39](#_Toc102205867)

[**Figure S58**. (+) HRESIMS Spectrum of Compound **6**	39](#_Toc102205868)

[**Figure S59**. UV Spectrum of Compound **6**	40](#_Toc102205869)

[**Figure S60**. IR (KBr disc) Spectrum of Compound **6**	40](#_Toc102205870)

[**Figure S61**. ^1^H NMR Spectrum of Compound **7** in CDCl_3_	41](#_Toc102205871)

[**Figure S62**. ^13^C NMR Spectrum of Compound **7** in CDCl_3_	41](#_Toc102205872)

[**Figure S63**. DEPT 135 Spectrum of Compound **7** in CDCl_3_	42](#_Toc102205873)

[**Figure S64**. ^1^H–^1^H COSY Spectrum of Compound **7** in CDCl_3_	42](#_Toc102205874)

[**Figure S65**. HSQC Spectrum of Compound **7** in CDCl_3_	43](#_Toc102205875)

[**Figure S66**. HMBC Spectrum of Compound **7** in CDCl_3_	43](#_Toc102205876)

[**Figure S67**. NOESY spectrum of compound **7** in CDCl_3_	44](#_Toc102205877)

[**Figure S68**. (+) HRESIMS Spectrum of Compound **7**	44](#_Toc102205878)

[**Figure S69**. UV Spectrum of Compound **7**	45](#_Toc102205879)

[**Figure S70**. IR (KBr disc) Spectrum of Compound **7**	45](#_Toc102205880)

[**Figure S71**. ^1^H NMR Spectrum of Compound **8** in CDCl_3_	46](#_Toc102205881)

[**Figure S72**. ^13^C NMR Spectrum of Compound **8** in CDCl_3_	46](#_Toc102205882)

[**Figure S73**. DEPT 135 Spectrum of Compound **8** in CDCl_3_	47](#_Toc102205883)

[**Figure S74**. ^1^H–^1^H COSY Spectrum of Compound **8** in CDCl_3_	47](#_Toc102205884)

[**Figure S75**. HSQC Spectrum of Compound **8** in CDCl_3_	48](#_Toc102205885)

[**Figure S76**. HMBC Spectrum of Compound **8** in CDCl_3_	48](#_Toc102205886)

[**Figure S77**. NOESY spectrum of compound **8** in CDCl_3_	49](#_Toc102205887)

[**Figure S78**. (+) HRESIMS Spectrum of Compound **8**	49](#_Toc102205888)

[**Figure S79**. UV Spectrum of Compound **8**	50](#_Toc102205889)

[**Figure S80**. IR (KBr disc) Spectrum of Compound **8**	50](#_Toc102205890)

[**Figure S81**. ^1^H NMR Spectrum of Compound **9** in CDCl_3_	51](#_Toc102205891)

[**Figure S82**. ^13^C NMR Spectrum of Compound **9** in CDCl_3_	51](#_Toc102205892)

[**Figure S83**. DEPT 135 Spectrum of Compound **9** in CDCl_3_	52](#_Toc102205893)

[**Figure S84**. ^1^H–^1^H COSY Spectrum of Compound **9** in CDCl_3_	52](#_Toc102205894)

[**Figure S85**. HSQC Spectrum of Compound **9** in CDCl_3_	53](#_Toc102205895)

[**Figure S86**. HMBC Spectrum of Compound **9** in CDCl_3_	53](#_Toc102205896)

[**Figure S87**. NOESY spectrum of compound **9** in CDCl_3_	54](#_Toc102205897)

[**Figure S88**. (+) HRESIMS Spectrum of Compound **9**	54](#_Toc102205898)

[**Figure S89**. UV Spectrum of Compound **9**	55](#_Toc102205899)

[**Figure S90**. IR (KBr disc) Spectrum of Compound **9**	55](#_Toc102205900)

[**Figure S91**. ^1^H NMR Spectrum of Compound **10** in CDCl_3_	56](#_Toc102205901)

[**Figure S92**. ^13^C NMR Spectrum of Compound **10** in CDCl_3_	56](#_Toc102205902)

[**Figure S93**. DEPT 135 Spectrum of Compound **10** in CDCl_3_	57](#_Toc102205903)

[**Figure S94**. ^1^H–^1^H COSY Spectrum of Compound **10** in CDCl_3_	57](#_Toc102205904)

[**Figure S95**. HSQC Spectrum of Compound **10** in CDCl_3_	58](#_Toc102205905)

[**Figure S96**. HMBC Spectrum of Compound **10** in CDCl_3_	58](#_Toc102205906)

[**Figure S97**. NOESY spectrum of compound **10** in CDCl_3_	59](#_Toc102205907)

[**Figure S98**. (+) HRESIMS Spectrum of Compound **10**	59](#_Toc102205908)

[**Figure S99**. UV Spectrum of Compound **10**	60](#_Toc102205909)

[**Figure S100**. IR (KBr disc) Spectrum of Compound **10**	60](#_Toc102205910)

[**Figure S101**. ^1^H NMR Spectrum of Compound **11** in CDCl_3_	61](#_Toc102205911)

[**Figure S102**. ^13^C NMR Spectrum of Compound **11** in CDCl_3_	61](#_Toc102205912)

[**Figure S103**. DEPT 135 Spectrum of Compound **11** in CDCl_3_	62](#_Toc102205913)

[**Figure S104**. ^1^H–^1^H COSY Spectrum of Compound **11** in CDCl_3_	62](#_Toc102205914)

[**Figure S105**. HSQC Spectrum of Compound **11** in CDCl_3_	63](#_Toc102205915)

[**Figure S106**. HMBC Spectrum of Compound **11** in CDCl_3_	63](#_Toc102205916)

[**Figure S107**. NOESY spectrum of compound **11** in CDCl_3_	64](#_Toc102205917)

[**Figure S108**. (+) HRESIMS Spectrum of Compound **11**	64](#_Toc102205918)

[**Figure S109**. UV Spectrum of Compound **11**	65](#_Toc102205919)

[**Figure S110**. IR (KBr disc) Spectrum of Compound **11**	65](#_Toc102205920)

[**Figure S111**. ^1^H NMR Spectrum of Compound **12** in CDCl_3_	66](#_Toc102205921)

[**Figure S112**. ^13^C NMR Spectrum of Compound **12** in CDCl_3_	66](#_Toc102205922)

[**Figure S113**. DEPT 135 Spectrum of Compound **12** in CDCl_3_	67](#_Toc102205923)

[**Figure S114**. ^1^H–^1^H COSY Spectrum of Compound **12** in CDCl_3_	67](#_Toc102205924)

[**Figure S115**. HSQC Spectrum of Compound **12** in CDCl_3_	68](#_Toc102205925)

[**Figure S116**. HMBC Spectrum of Compound **12** in CDCl_3_	68](#_Toc102205926)

[**Figure S117**. NOESY spectrum of compound **12** in CDCl_3_	69](#_Toc102205927)

[**Figure S118**. (+) HRESIMS Spectrum of Compound **12**	69](#_Toc102205928)

[**Figure S119**. UV Spectrum of Compound **12**	70](#_Toc102205929)

[**Figure S120**. IR (KBr disc) Spectrum of Compound **12**	70](#_Toc102205930)

[**Figure S121**. ECD Spectrum of **1**–**6**	71](#_Toc102205931)

[**Figure S122**. ECD Spectrum of **7**–**12**	71](#_Toc102205932)

**Table S1**. Crystal data and structure refinement for Compound **6**

| Identification code | 6 |
| --- | --- |
| Empirical formula | C_19_H_24_O_6_ |
| Formula weight | 348.38 |
| Temperature/K | 170.00(10) |
| Crystal system | orthorhombic |
| Space group | P2_1_2_1_2_1_ |
| a/Å | 8.6975(2) |
| b/Å | 9.7500(2) |
| c/Å | 20.3304(5) |
| α/° | 90 |
| β/° | 90 |
| γ/° | 90 |
| Volume/Å^3^ | 1724.03(7) |
| Z | 4 |
| ρcalcg/cm^3^ | 1.342 |
| μ/mm^‑1^ | 0.823 |
| F(000) | 744.0 |
| Crystal size/mm^3^ | 0.14 × 0.12 × 0.1 |
| Radiation | Cu Kα (λ = 1.54184) |
| 2Θ range for data collection/° | 8.698 to 147.768 |
| Index ranges | -10 ≤ h ≤ 7, -11 ≤ k ≤ 11, -24 ≤ l ≤ 23 |
| Reflections collected | 9848 |
| Independent reflections | 3423 [R_int_ = 0.0325, R_sigma_ = 0.0320] |
| Data/restraints/parameters | 3423/0/230 |
| Goodness-of-fit on F^2^ | 1.047 |
| Final R indexes [I>=2σ (I)] | R_1_ = 0.0356, wR_2_ = 0.0887 |
| Final R indexes [all data] | R_1_ = 0.0386, wR_2_ = 0.0914 |
| Largest diff. peak/hole / e Å^-3^ | 0.26/-0.23 |
| Flack parameter | -0.04(9) |
|  |  |
|  |  |
|  |  |
|  |  |
|  |  |
|  |  |
|  |  |

**Table S1**. Crystal data and structure refinement for Compound **8**

| Identification code | 8 |
| --- | --- |
| Empirical formula | C_19_H_24_O_6_ |
| Formula weight | 348.38 |
| Temperature/K | 179.99(10) |
| Crystal system | orthorhombic |
| Space group | P2_1_2_1_2_1_ |
| a/Å | 9.34750(10) |
| b/Å | 10.61860(10) |
| c/Å | 17.3505(2) |
| α/° | 90 |
| β/° | 90 |
| γ/° | 90 |
| Volume/Å^3^ | 1722.16(3) |
| Z | 4 |
| ρ_calc_g/cm^3^ | 1.344 |
| μ/mm^‑1^ | 0.824 |
| F(000) | 744.0 |
| Crystal size/mm^3^ | 0.15 × 0.13 × 0.12 |
| Radiation | Cu Kα (λ = 1.54184) |
| 2Θ range for data collection/° | 9.766 to 147.578 |
| Index ranges | -11 ≤ h ≤ 11, -13 ≤ k ≤ 13, -18 ≤ l ≤ 21 |
| Reflections collected | 18414 |
| Independent reflections | 3462 [R_int_ = 0.0271, R_sigma_ = 0.0160] |
| Data/restraints/parameters | 3462/0/239 |
| Goodness-of-fit on F^2^ | 1.090 |
| Final R indexes [I>=2σ (I)] | R_1_ = 0.0293, wR_2_ = 0.0772 |
| Final R indexes [all data] | R_1_ = 0.0297, wR_2_ = 0.0776 |
| Largest diff. peak/hole / e Å^-3^ | 0.18/-0.17 |
| Flack parameter | 0.01(5) |

**Table S3**. Crystal data and structure refinement for Compound **10**

| Identification code | 10 |
| --- | --- |
| Empirical formula | C_19_H_24_O_6_ |
| Formula weight | 348.38 |
| Temperature/K | 169.99(10) |
| Crystal system | monoclinic |
| Space group | P2_1_ |
| a/Å | 8.4422(4) |
| b/Å | 7.1913(4) |
| c/Å | 15.2596(6) |
| α/° | 90 |
| β/° | 99.791(4) |
| γ/° | 90 |
| Volume/Å^3^ | 912.92(8) |
| Z | 2 |
| ρ_calc_g/cm^3^ | 1.267 |
| μ/mm^‑1^ | 0.777 |
| F(000) | 372.0 |
| Crystal size/mm^3^ | 0.13 × 0.11 × 0.1 |
| Radiation | Cu Kα (λ = 1.54184) |
| 2Θ range for data collection/° | 5.878 to 148.026 |
| Index ranges | -8 ≤ h ≤ 10, -8 ≤ k ≤ 8, -18 ≤ l ≤ 18 |
| Reflections collected | 10067 |
| Independent reflections | 3411 [R_int_ = 0.0440, R_sigma_ = 0.0498] |
| Data/restraints/parameters | 3411/1/242 |
| Goodness-of-fit on F^2^ | 1.053 |
| Final R indexes [I>=2σ (I)] | R_1_ = 0.0445, wR_2_ = 0.1059 |
| Final R indexes [all data] | R_1_ = 0.0576, wR_2_ = 0.1126 |
| Largest diff. peak/hole / e Å^-3^ | 0.14/-0.15 |
| Flack parameter | -0.08(15) |

**Table S4** Crystal data and structure refinement for Compound **12**

| Identification code | 12 |
| --- | --- |
| Empirical formula | C_15_H_19_ClO_5_ |
| Formula weight | 314.75 |
| Temperature/K | 200.00(10) |
| Crystal system | orthorhombic |
| Space group | P2_1_2_1_2_1_ |
| a/Å | 5.80960(10) |
| b/Å | 14.6880(2) |
| c/Å | 16.6063(2) |
| α/° | 90 |
| β/° | 90 |
| γ/° | 90 |
| Volume/Å^3^ | 1417.04(4) |
| Z | 4 |
| ρ_calc_g/cm^3^ | 1.475 |
| μ/mm^‑1^ | 2.575 |
| F(000) | 664.0 |
| Crystal size/mm^3^ | 0.14 × 0.12 × 0.11 |
| Radiation | Cu Kα (λ = 1.54184) |
| 2Θ range for data collection/° | 8.036 to 147.536 |
| Index ranges | -6 ≤ h ≤ 7, -18 ≤ k ≤ 18, -20 ≤ l ≤ 20 |
| Reflections collected | 14845 |
| Independent reflections | 2827 [R_int_ = 0.0403, R_sigma_ = 0.0232] |
| Data/restraints/parameters | 2827/0/266 |
| Goodness-of-fit on F^2^ | 1.090 |
| Final R indexes [I>=2σ (I)] | R_1_ = 0.0300, wR_2_ = 0.0758 |
| Final R indexes [all data] | R_1_ = 0.0304, wR_2_ = 0.0760 |
| Largest diff. peak/hole / e Å^-3^ | 0.19/-0.25 |
| Flack parameter | 0.002(7) |

**Figure S1**. ^1^H NMR Spectrum of Compound **1** in CDCl_3_

**Figure S2**. ^13^C NMR Spectrum of Compound **1** in CDCl_3_

**Figure S3**. DEPT 135 Spectrum of Compound **1** in CDCl_3_

**Figure S4**. ^1^H–^1^H COSY Spectrum of Compound **1** in CDCl_3_

**Figure S5**. HSQC Spectrum of Compound **1** in CDCl_3_

**Figure S6**. HMBC Spectrum of Compound **1** in CDCl_3_

**Figure S7**. NOESY spectrum of compound **1** in CDCl_3_

**Figure S8**. (+) HRESIMS Spectrum of Compound **1**

**Figure S9**. UV Spectrum of Compound **1**

**Figure S10**. IR (KBr disc) Spectrum of Compound **1**

**Figure S11**. ^1^H NMR Spectrum of Compound **2** in CDCl_3_

**Figure S12**. ^13^C NMR Spectrum of Compound **2** in CDCl_3_

**Figure S13**. DEPT 135 Spectrum of Compound **2** in CDCl_3_

**Figure S14**. ^1^H–^1^H COSY Spectrum of Compound **2** in CDCl_3_

**Figure S15**. HSQC Spectrum of Compound **2** in CDCl_3_

**Figure S16**. HMBC Spectrum of Compound **2** in CDCl_3_

**Figure S17**. NOESY spectrum of compound **2** in CDCl_3_

**Figure S18**. (+) HRESIMS Spectrum of Compound **2**

**Figure S19**. UV Spectrum of Compound **2**

**Figure S20**. IR (KBr disc) Spectrum of Compound **2**

**Figure S21**. ^1^H NMR Spectrum of Compound **3** in CDCl_3_

**Figure S22**. ^13^C NMR Spectrum of Compound **3** in CDCl_3_

**Figure S23**. DEPT 135 Spectrum of Compound **3** in CDCl_3_

**Figure S24**. ^1^H–^1^H COSY Spectrum of Compound **3** in CDCl_3_

**Figure S25**. HSQC Spectrum of Compound **3** in CDCl_3_

**Figure S26**. HMBC Spectrum of Compound **3** in CDCl_3_

**Figure S27**. NOESY spectrum of compound **3** in CDCl_3_

**Figure S28**. (+) HRESIMS Spectrum of Compound **3**

**Figure S29**. UV Spectrum of Compound **3**

**Figure S30**. IR (KBr disc) Spectrum of Compound **3**

**Figure S31**. ^1^H NMR Spectrum of Compound **4** in CDCl_3_

**Figure S32**. ^13^C NMR Spectrum of Compound **4** in CDCl_3_

**Figure S33**. DEPT 135 Spectrum of Compound **4** in CDCl_3_

**Figure S34**. ^1^H–^1^H COSY Spectrum of Compound **4** in CDCl_3_

**Figure S35**. HSQC Spectrum of Compound **4** in CDCl_3_

**Figure S36**. HMBC Spectrum of Compound **4** in CDCl_3_

**Figure S37**. NOESY spectrum of compound **4** in CDCl_3_

**Figure S38**. (+) HRESIMS Spectrum of Compound **4**

**Figure S39**. UV Spectrum of Compound **4**

**Figure S40**. IR (KBr disc) Spectrum of Compound **4**

**Figure S41**. ^1^H NMR Spectrum of Compound **5** in CDCl_3_

**Figure S42**. ^13^C NMR Spectrum of Compound **5** in CDCl_3_

**Figure S43**. DEPT 135 Spectrum of Compound **5** in CDCl_3_

**Figure S44**. ^1^H–^1^H COSY Spectrum of Compound **5** in CDCl_3_

**Figure S45**. HSQC Spectrum of Compound **5** in CDCl_3_

**Figure S46**. HMBC Spectrum of Compound **5** in CDCl_3_

**Figure S47**. NOESY spectrum of compound **5** in CDCl_3_

**Figure S48**. (+) HRESIMS Spectrum of Compound **5**

**Figure S49**. UV Spectrum of Compound **5**

**Figure S50**. IR (KBr disc) Spectrum of Compound **5**

**Figure S51**. ^1^H NMR Spectrum of Compound **6** in CDCl_3_

**Figure S52**. ^13^C NMR Spectrum of Compound **6** in CDCl_3_

**Figure S53**. DEPT 135 Spectrum of Compound **6** in CDCl_3_

**Figure S54**. ^1^H–^1^H COSY Spectrum of Compound **6** in CDCl_3_

**Figure S55**. HSQC Spectrum of Compound **6** in CDCl_3_

**Figure S56**. HMBC Spectrum of Compound **6** in CDCl_3_

**Figure S57**. NOESY spectrum of compound **6** in CDCl_3_

**Figure S58**. (+) HRESIMS Spectrum of Compound **6**

**Figure S59**. UV Spectrum of Compound **6**

**Figure S60**. IR (KBr disc) Spectrum of Compound **6**

**Figure S61**. ^1^H NMR Spectrum of Compound **7** in CDCl_3_

**Figure S62**. ^13^C NMR Spectrum of Compound **7** in CDCl_3_

**Figure S63**. DEPT 135 Spectrum of Compound **7** in CDCl_3_

**Figure S64**. ^1^H–^1^H COSY Spectrum of Compound **7** in CDCl_3_

**Figure S65**. HSQC Spectrum of Compound **7** in CDCl_3_

**Figure S66**. HMBC Spectrum of Compound **7** in CDCl_3_

**Figure S67**. NOESY spectrum of compound **7** in CDCl_3_

**Figure S68**. (+) HRESIMS Spectrum of Compound **7**

**Figure S69**. UV Spectrum of Compound **7**

**Figure S70**. IR (KBr disc) Spectrum of Compound **7**

**Figure S71**. ^1^H NMR Spectrum of Compound **8** in CDCl_3_

**Figure S72**. ^13^C NMR Spectrum of Compound **8** in CDCl_3_

**Figure S73**. DEPT 135 Spectrum of Compound **8** in CDCl_3_

**Figure S74**. ^1^H–^1^H COSY Spectrum of Compound **8** in CDCl_3_

**Figure S75**. HSQC Spectrum of Compound **8** in CDCl_3_

**Figure S76**. HMBC Spectrum of Compound **8** in CDCl_3_

**Figure S77**. NOESY spectrum of compound **8** in CDCl_3_

**Figure S78**. (+) HRESIMS Spectrum of Compound **8**

**Figure S79**. UV Spectrum of Compound **8**

**Figure S80**. IR (KBr disc) Spectrum of Compound **8**

**Figure S81**. ^1^H NMR Spectrum of Compound **9** in CDCl_3_

**Figure S82**. ^13^C NMR Spectrum of Compound **9** in CDCl_3_

**Figure S83**. DEPT 135 Spectrum of Compound **9** in CDCl_3_

**Figure S84**. ^1^H–^1^H COSY Spectrum of Compound **9** in CDCl_3_

**Figure S85**. HSQC Spectrum of Compound **9** in CDCl_3_

**Figure S86**. HMBC Spectrum of Compound **9** in CDCl_3_

**Figure S87**. NOESY spectrum of compound **9** in CDCl_3_

**Figure S88**. (+) HRESIMS Spectrum of Compound **9**

**Figure S89**. UV Spectrum of Compound **9**

**Figure S90**. IR (KBr disc) Spectrum of Compound **9**

**Figure S91**. ^1^H NMR Spectrum of Compound **10** in CDCl_3_

**Figure S92**. ^13^C NMR Spectrum of Compound **10** in CDCl_3_

**Figure S93**. DEPT 135 Spectrum of Compound **10** in CDCl_3_

**Figure S94**. ^1^H–^1^H COSY Spectrum of Compound **10** in CDCl_3_

**Figure S95**. HSQC Spectrum of Compound **10** in CDCl_3_

**Figure S96**. HMBC Spectrum of Compound **10** in CDCl_3_

**Figure S97**. NOESY spectrum of compound **10** in CDCl_3_

**Figure S98**. (+) HRESIMS Spectrum of Compound **10**

**Figure S99**. UV Spectrum of Compound **10**

**Figure S100**. IR (KBr disc) Spectrum of Compound **10**

**Figure S101**. ^1^H NMR Spectrum of Compound **11** in CDCl_3_

**Figure S102**. ^13^C NMR Spectrum of Compound **11** in CDCl_3_

**Figure S103**. DEPT 135 Spectrum of Compound **11** in CDCl_3_

**Figure S104**. ^1^H–^1^H COSY Spectrum of Compound **11** in CDCl_3_

**Figure S105**. HSQC Spectrum of Compound **11** in CDCl_3_

**Figure S106**. HMBC Spectrum of Compound **11** in CDCl_3_

**Figure S107**. NOESY spectrum of compound **11** in CDCl_3_

**Figure S108**. (+) HRESIMS Spectrum of Compound **11**

**Figure S109**. UV Spectrum of Compound **11**

**Figure S110**. IR (KBr disc) Spectrum of Compound **11**

**Figure S111**. ^1^H NMR Spectrum of Compound **12** in CDCl_3_

**Figure S112**. ^13^C NMR Spectrum of Compound **12** in CDCl_3_

**Figure S113**. DEPT 135 Spectrum of Compound **12** in CDCl_3_

**Figure S114**. ^1^H–^1^H COSY Spectrum of Compound **12** in CDCl_3_

**Figure S115**. HSQC Spectrum of Compound **12** in CDCl_3_

**Figure S116**. HMBC Spectrum of Compound **12** in CDCl_3_

**Figure S117**. NOESY spectrum of compound **12** in CDCl_3_

**Figure S118**. (+) HRESIMS Spectrum of Compound **12**

**Figure S119**. UV Spectrum of Compound **12**

**Figure S120**. IR (KBr disc) Spectrum of Compound **12**

**Figure S121**. ECD Spectrum of **1**–**6**

**Figure S122**. ECD Spectrum of **7**–**12**
